# Determining optimal practices for foal weaning – A protocol for a systematic review and network meta-analysis

**DOI:** 10.1371/journal.pone.0352182

**Published:** 2026-07-01

**Authors:** Nicole Cranston, Petra Buckley, Lorraine Rose, Jaymie Loy, Hayley Randle

**Affiliations:** 1 Faculty of Science and Health, School of Agricultural, Environmental and Veterinary Sciences, Charles Sturt University, Wagga Wagga, New South Wales, Australia; 2 Faculty of Science and Health Library Team, Library Services, Charles Sturt University, Port Macquarie, New South Wales, Australia; University of Life Sciences in Lublin, POLAND

## Abstract

**Background:**

Weaning is a stressful time in a foal’s (*Equus caballus*) life. As humans artificially manage weaning in domestic horses, it provides an opportunity to safeguard horse welfare early in life, with potential long-term benefits. While numerous studies have examined the effects of different weaning and management interventions on foal stress, optimal weaning practices have yet to be identified. **Overall purpose of the study:** To identify which weaning interventions and management practices have the greatest potential to improve foal welfare based on physiological and behavioural outcomes. **Research questions:** (1) What physiological and behavioural changes occur in foals in response to artificial weaning? (2) Do different interventions during weaning produce different physiological and behavioural outcomes in foals? (3) How do study-level characteristics explain heterogeneity in foal outcomes during weaning, and which impact welfare outcomes across different interventions? (4) Which interventions, management practices, or combinations thereof most effectively optimise foal welfare during weaning?

**Methodology:**

Six databases were searched on 24 August 2025. Database search strings were based on the Population-Intervention-Comparator-Outcome (PICO) framework and were PRESS-reviewed. Additional studies, including grey literature, will be identified through dissertation databases, ScienceDirect, Google Scholar, and by hand-searching the reference lists of included studies and narrative reviews, to ensure a comprehensive search. Articles will be selected based on predefined eligibility criteria. Two reviewers will independently conduct title/abstract and full-text screening, data extraction, and assess risk of bias and confidence in the evidence (GRADE and CINeMA). Results will be synthesised through pairwise and Bayesian network meta-analysis using R Statistical Software.

**Conclusion:**

This systematic review will be the first to investigate foal welfare during artificial weaning, and the findings are expected to provide evidence-based recommendations to improve foal welfare. This paper outlines the protocol that will be used and does not contain any empirical results.

**Registration:**

This systematic review protocol was registered on the Open Science Framework (OSF) on 19 May 2025 (https://doi.org/10.17605/OSF.IO/DJWQY).

## Introduction

The horse industry faces increasing public pressure to improve equine welfare [1,2]. The growing public scrutiny of equine welfare, evidenced by increased media coverage and consumer surveys [3,4] have placed pressure on the industry to improve the ethical treatment of horses (*Equus caballus*) [2]. The equine industry needs to optimise equine welfare through ethical practices to retain public trust, ensure its long-term viability, and secure its social licence to operate [1,2,5,6]. While scrutiny often focuses on elite equestrian sports, broader welfare considerations of equine management processes, such as weaning, are also gaining attention [7]. Since foals across all equine sectors are artificially weaned [8], evaluating and modifying practices during this critical life stage is expected to significantly enhance horse welfare.

Weaning is recognised as one of the most stressful events in a horse’s life [9–12]. As with other mammals, weaning is defined as the nutritional separation from the mare [11,13]. In naturalistic settings, weaning usually occurs spontaneously around nine months of age, with foals remaining with their maternal herd until they are two to three years old [11]. In domestic settings, however, weaning is usually artificially implemented and generally occurs between four and eight months of age [9–11,14,15]. In addition to nutritional separation, weaning also involves complete physical, visual, and auditory separation of the foal from the mare. The splitting of the mare and foal dyad is defined as either abrupt weaning, due to immediate separation, or gradual weaning if separation occurs in stages. Weaning may also occur in an unfamiliar environment and often involves individual foals kept in complete isolation. Domestic weaning therefore involves not only nutritional separation but also the disruption of strong social bonds, contributing to significant psychological stress in foals, which is further compounded in those weaned abruptly.

The choice of weaning method has historically been based on manager preference [16] and site facilities [9]. Foals subjected to artificial weaning demonstrate several physiological and behavioural stress responses, including weight loss, increased cortisol levels, vocalisation, and locomotion [9,10,17]. When foals are weaned using gradual methods, some studies have reported fewer physiological and behavioural stress indicators. However, the definitions of gradual weaning vary widely, and few studies clearly specify when they define the weaning process to have commenced. In addition, many studies do not adequately control for potential confounding factors such as foal age, housing, and the presence of conspecifics during weaning. As a result of this study-level heterogeneity, comparisons between studies and weaning methods are difficult, and the extent of any observed welfare benefits remains unclear.

Other management strategies to reduce weaning stress, including weaning with foal conspecifics or ‘nannies’ (adult horse/s) [18–22], modifying pre- and post-weaning diets [18,23–26], weaning at a later age [27], or using pharmacological interventions [28–30] may potentially be incorporated into weaning interventions to further enhance welfare. Physical and social environmental factors, such as housing and social changes during weaning, can even contribute to stress [11,31,32], and should be considered when planning a weaning program.

There can be long-term consequences of the stress experienced by foals during weaning. Reduced weight gain and performance [33,34], as well as increased prevalence of stereotypies [14,35,36] have been associated with weaning. In many mammal species, acute and chronic stress early in life has been shown to affect neuropsychological development, altering the function of the nervous, endocrine, and immune systems, and negatively impacting emotional, social, and cognitive development [7,34]. This, in turn, can affect learning ability, fearfulness, and handling efficiency which influence performance, and ultimately the commercial value of the horse [37–41]. Therefore, it is in the industry’s best interest to adopt weaning practices that safeguard foal welfare.

Increasing societal pressure for animals to be free from distress, with opportunities to experience positive emotional states [42] has created a need to analyse and refine industry practices to improve welfare. Although weaning will inevitably result in some degree of stress, there is still no clear consensus on how to best minimise its impact. The current evidence is inconsistent, fragmented, and difficult to interpret, making it challenging for the industry to identify and adopt optimal practices. While one scoping and several narrative reviews have described and compared equine weaning practices [9–12,17], no formal synthesis has been undertaken. As the equine industry moves towards welfare-focused practices [3,43], there is a need to synthesise existing knowledge to appraise current evidence and identify knowledge gaps, to enable the breeding industry to advance towards optimal weaning strategies.

A systematic review is a standard methodology for critically appraising and synthesising evidence to provide industries with evidence-based best-practice recommendations [44,45]. This systematic review protocol is proposed to critically evaluate the available literature on foal weaning. A pairwise meta-analysis will be used to determine the physiological and behavioural changes associated with weaning. Additionally, as weaning is conceptually a complex intervention, network meta-analysis (NMA) will be conducted, as it allows the synthesis of all the available evidence, combining direct and indirect evidence, allowing for the comparison of multiple interventions and enabling practices to be ranked to assist with decision-making regarding foal welfare optimal practices [46].

### Objectives

The aim of the systematic review is to critically evaluate the physiological and behavioural outcomes of artificial weaning in equine foals and will identify which weaning and management interventions most effectively safeguard foal welfare. In addition to comparing interventions, the review will examine study-level characteristics (foal age, sex, housing environment, presence of conspecifics during weaning, and time since weaning) as contextual variables, which may act as moderating factors influencing intervention effects, or design-related variables (subgroups), which may account for between-study heterogeneity.

The first objective will assess the overall effects of artificial weaning on foal physiology and behaviour. This objective will provide a broad overview of the general impact of artificial weaning, identifying consistent physiological and behavioural patterns, irrespective of weaning method, through random-effects pairwise meta-analysis. The specific research question is: *what physiological and behavioural changes occur in foals in response to artificial weaning?*

The second objective will estimate and compare the effects of the different weaning and management interventions on foal physiological and behavioural outcomes. This objective will examine whether certain intervention types are associated with more- or less-pronounced stress responses or improved welfare effects, using Bayesian NMA. The specific research question is: *do different interventions during weaning produce different physiological and behavioural outcomes in foals?*

The third objective will examine how pre-defined study-level characteristics contribute to variability in weaning outcomes and will identify which factors moderate or influence the effects of weaning. This objective will explore these characteristics as potential sources of heterogeneity or modifying factors across trials using component-based Bayesian NMA. Assessing how these characteristics modify physiological and behavioural outcomes, and how they interact with intervention effects, will refine the interpretation of results and highlight the conditions under which specific interventions are more- or less-effective. The specific research question is: *how do study-level characteristics explain heterogeneity in foal outcomes during weaning, and which impact welfare outcomes across different interventions?*

Finally, the fourth objective will identify which intervention or management practices improve foal welfare during weaning. Probability ranking of the Bayesian NMA results will be used to inform which practices support positive welfare outcomes and provide evidence-based recommendations for industry and future research. The specific research question is: *which interventions, management practices or combination thereof, most effectively optimise foal welfare during weaning?*

## Methods

The systematic review protocol was developed *a priori* and registered on the Open Science Framework (OSF; https://osf.io) on 19 May 2025 (https://doi.org/10.17605/OSF.IO/DJWQY). It was prepared in accordance with the PRISMA-P statement [47,48] and PRISMA-NMA checklist [49] (see S1 Checklist), guided by Cochrane’s recommendations for systematic review design and execution [44]. Any amendments to the protocol will be documented and justified in a table of adjustments and added to the registration site upon publication of the completed review [50].

### Eligibility criteria

The eligibility criteria were developed collaboratively by the review authors based on the Population-Intervention-Comparator-Outcome (PICO) framework [51].

#### Population.

The population of interest will be *Equus caballus* foals of any breed. The population will be restricted to foals under one year of age, as the equine industry rarely weans foals beyond this age [11,15]. Studies of neonate foals (<one month of age), individuals weaned at or after one year of age, non-*Equus caballus* species, and those on young or adult horses (≥ 1 year of age) will be excluded. Studies that assess both the target and excluded populations will be included only if data for the target population are reported separately. For this review, it will be assumed that any foal who meets the inclusion criteria will, in principle, be equally likely to be allocated to any eligible intervention.

#### Interventions.

All studies containing planned artificial weaning or separations of the target population will be included. Weaning in this review will be defined as the commencement of permanent nutritional and physical separation of the foal from the dam [10]. Abrupt weaning will be characterised as the immediate and complete separation of the foal from the dam without prior acclimatisation. This may occur individually (reference intervention), in pairs or in groups of foals, but without the presence of other adults. The reference intervention will be compared to alternative *weaning interventions*, including:

1) Abrupt weaning with a nanny (adult horse): foal weaned abruptly from the dam but left in the care of another adult horse;
2) Progressive removal of mares: progressive removal of 1–2 dams from an established dam-foal herd through the abrupt weaning of the foal over ≥ 2 days until all mares have been removed;
3) Gradual acclimatisation: foals gradually acclimatised to separations prior to (≥ 2 weeks) the commencement of the weaning process;
4) Gradual partial contact: partial contact maintained for ≥ 2 days prior to complete separation (e.g., fenceline, nutritional separation via covering udder);
5) Gradual over time: increasing separation time ≥ 2 days prior to complete separation;
6) Gradual over distance: increasing the distance separated over ≥ 2 days prior to complete separation; and
7) Other: any other weaning intervention not covered by previous definitions.

Any additional management strategy specifically explored at the study treatment level, while keeping the primary weaning intervention constant, will be classified as a *management intervention*. The *management interventions* will be categorised as:

- Environmental (housing, conspecifics, handling)
- Temporal (age at weaning)
- Dietary (pre/post-weaning nutrition)
- Medical (pharmaceutical use).

However, when these factors are observed but not evaluated as a treatment-level comparison, they will be considered study-level characteristics and explored as subgroup or moderating factors. Additionally, experiments reporting temporary separations of the target population from their dam, with a management intervention aimed at reducing stress during the separation will also be included.

Instances where weaning is unplanned, such as due to the mare’s death or emergency health interventions, will be excluded, as will research involving the target population if the study commenced only after the foal was weaned, or the study was not related at all to weaning or separation. Observations of weaning in wild/feral equine populations will also be excluded, as they do not reflect domestic weaning practices.

#### Comparisons.

All studies assessing weaning will be included if pre- and post-weaning data are reported, regardless of whether a direct comparator group is included. Comparisons may include:

1) Control group (unweaned) versus any form of weaning or separation
2) Abrupt weaning versus any alternative weaning intervention
3) Comparison between two alternative weaning interventions
4) Comparison between management interventions within the same weaning intervention
5) Temporary separation with a management intervention aimed at reducing stress
6) Any intervention with pre- and post-intervention comparisons

#### Primary outcomes.

Articles will not be excluded based on the outcomes reported [51]. Primary outcomes of interest will include physiological and behavioural indicators measured by independent assessors (Table 1). Outcomes will be extracted as reported (e.g., dichotomous, continuous). Due to potential variation in behavioural definitions, these will also be extracted as reported.

**Table 1:** Primary and secondary outcomes.

	Physiological	Behavioural
**Primary** **Outcomes**	Cortisol	Locomotion
	Heart rate	Vocalisation
	Body weight	Agonistic behaviour
		Defecation
		Eating
**Secondary outcomes**	Any other physiological outcome with sufficient studies for comparison	Any other behavioural outcome with sufficient studies for comparison

#### Study design.

The review will include randomised controlled trials (RCTs), cluster-RCTs, non-randomised trials (non-RCTs), controlled before-and-after (CBA) trials, prospective and retrospective comparative cohort studies, cross-sectional, case-series, and observational studies in the narrative synthesis. Inclusion of these study designs will ensure a comprehensive assessment of the available evidence where high-quality comparative studies are limited. Narrative synthesis without meta-analysis will only be undertaken if higher-quality evidence is insufficient [52]. Questionnaires, individual case reports, narrative and scoping reviews, will be excluded, as will superseded book or chapter editions, news reports, editorials and opinion pieces. Only RCTs, cluster-RCTs, non-RCTs, and CBA trials will be included in meta-analyses.

#### Reporting characteristics.

There will be no limitations based on the year of publication, geographical location, publication type, or language. Articles not written in English will be translated where possible, and those that cannot be translated will be excluded from full-text screening.

#### Exclusion criteria hierarchy.

The reason for exclusion will be reported based on the following hierarchical order:

1) Population – non-*Equus caballus* species
2) Population – individuals weaned after > 1 year of age
3) Population – neonate foals (≤ 1 month of age)
4) Population – young or adult (including mares) horses (> 1 year of age)
5) Intervention – unplanned weaning (e.g., mare death or emergency health intervention)
6) Intervention – includes target population but data collection commenced only after the foal was weaned
7) Intervention – on target population study not related to weaning or separation
8) Intervention – weaning of foals in wild/feral equine populations
9) Comparator – no included comparator group
10) Study types – narrative and scoping reviews, questionnaires, individual case reports, superseded book or chapter editions, new reports, editorials and opinion pieces
11) English translation unavailable.

### Information sources

To maximise the likelihood of locating all articles on a topic (recall), it is recommended that at least four databases be searched, including Web of Science and Google Scholar [53]. For this review, six databases will be searched: ProQuest One Academic (ProQuest), CAB Abstracts (Ovid), PsycINFO (Ovid), PubMed (National Library of Medicine), Scopus (Elsevier), and Web of Science (Clarivate). The initial bibliographical database literature search was conducted by the primary author on 27 August 2025.

Grey literature will also be included in the search strategy to reduce publication bias [54]. To be as comprehensive as possible, Open Access Theses and Dissertations (OATD), EBSCO Open Dissertations, International Veterinary Information Services (IVIS), SciQuest, and Science Direct will be searched, and the first 200 references (sorted by relevance) identified in Google Scholar will be included, as relevance declines rapidly, and this approach is recommended to balance sensitivity with feasibility [53,55]. Relevant industry organisations and researchers will be contacted regarding any unpublished reports [56], in addition to hand-searching reference lists of topical literature reviews and included primary articles. Despite these measures, some degree of publication bias may remain, and conducting the search in English may limit the retrieval of relevant studies published in other languages. The search will be repeated immediately prior to data synthesis to identify any new research and again if the review is not published within 12 months of the previous search. Any studies published after this time will be noted but excluded from the synthesis.

### Search strategy

The PICO framework guided the selection of search terms. In accordance with Cochrane recommendations, search terms were restricted to population and intervention [56]. The sensitivity of the search strategy was pilot tested by NC through the retrieval of known items [57] in CAB Abstracts, using five studies that had been identified *a priori* [10,11,27,58,59]. All articles were successfully retrieved. The search strategy was subsequently translated for use in the other databases and independently validated by LR using the Peer Review of Electronic Search Strategies (PRESS) checklist to ensure search precision [60]. The full search process will be documented using PRISMA-S (Search extension) and will be uploaded to OSF to enhance reproducibility [61].

The following search terms were selected: “foal* OR (equi* OR horse) weanling* (within three words)” AND “weaning OR wean OR separation”. Boolean operators were adapted for each bibliographical database to balance sensitivity and specificity [53], and the terms were combined with filters for title, abstract, and subject fields (Table 2).

**Table 2:** Bibliographical database search strategy.

Database	Population	Intervention
		AND
CAB Abstracts^1^	foal/ OR (foal* OR (equi* adj3 weanling*)).mp. OR (horse adj3 weanling*).ab,ti.	weaning/ OR (weaning OR wean OR separation).ab,ti
		AND
ProQuest One Academic	title((foal* OR (equi* NEAR/3 weanling*) OR (horse NEAR/3 weanling*))) OR abstract((foal* OR (equi* NEAR/3 weanling*) OR (horse NEAR/3 weanling*))) OR subject((foal* OR (equi* NEAR/3 weanling*) OR (horse NEAR/3 weanling*)))	title((wean OR weaning OR separation)) OR abstract((wean OR weaning OR separation)) OR subject((wean OR weaning OR separation))
		AND
PsycInfo^2^	(foal* OR (equi* adj3 weanling*)).mp. OR (horse adj3 weanling).ab,sh,ti.	(weaning or wean or separation).ab,sh,ti.
		AND
PubMed	(foal*[Title/Abstract]) OR (“horse weanlings”[Title/Abstract: ~ 3]) OR (“horse weanling”[Title/Abstract: ~ 3]) OR (“equine weanling”[Title/Abstract: ~ 3]) OR (“equine weanlings”[Title/Abstract: ~ 3])	(weaning[MeSH Terms]) OR (wean[Title/Abstract] OR weaning[Title/Abstract] OR separation[Title/Abstract])
		AND
Scopus	TITLE-ABS-KEY (foal* OR (equi* W/3 weanling*) OR (horse W/3 weanling*))	TITLE-ABS-KEY (wean OR weaning OR separation)
		AND
Web of Science	foal* OR (equi* NEAR/3 weanling*) OR (horse NEAR/3 weanling*) (Title) or foal* OR (equi* NEAR/3 weanling*) OR (horse NEAR/3 weanling*) (Abstract)	Wean OR weaning OR separation (Title) or Wean OR weaning OR separation (Abstract)

^1^ / = subject headings; [mp = abstract(ab), title(ti), original title, heading words, cabicodes words].

^2^ sh = subject heading; [mp = title(ti), abstract(ab), heading word, table of contents, key concepts, original title, tests & measures, mesh word].

### Study records

#### Data management.

All search result citations will be imported and managed in Covidence (https://www.covidence.org/), a web-based systematic review management tool that facilitates independence and collaboration among reviewers during study selection, data extraction and risk of bias (RoB) assessment [62]. Articles that cannot be directly imported into Covidence will be imported via EndNote^TM^21 (Clarivate Analytics, Philadelphia, PA, USA). Covidence automatically detects and removes duplicate records based on title, year, volume, and author(s). Any duplicate publications identified during screening will be manually checked using the same criteria and reported separately in the PRISMA flow diagram. Multiple reports of the same study will be cross-checked against authorship, start date and duration, population characteristics and sample size, location, ethics approval numbers, and registration numbers, and will be merged, with the most comprehensive report designated as the primary data source for reporting purposes.

#### Selection process.

**Fig 1:**
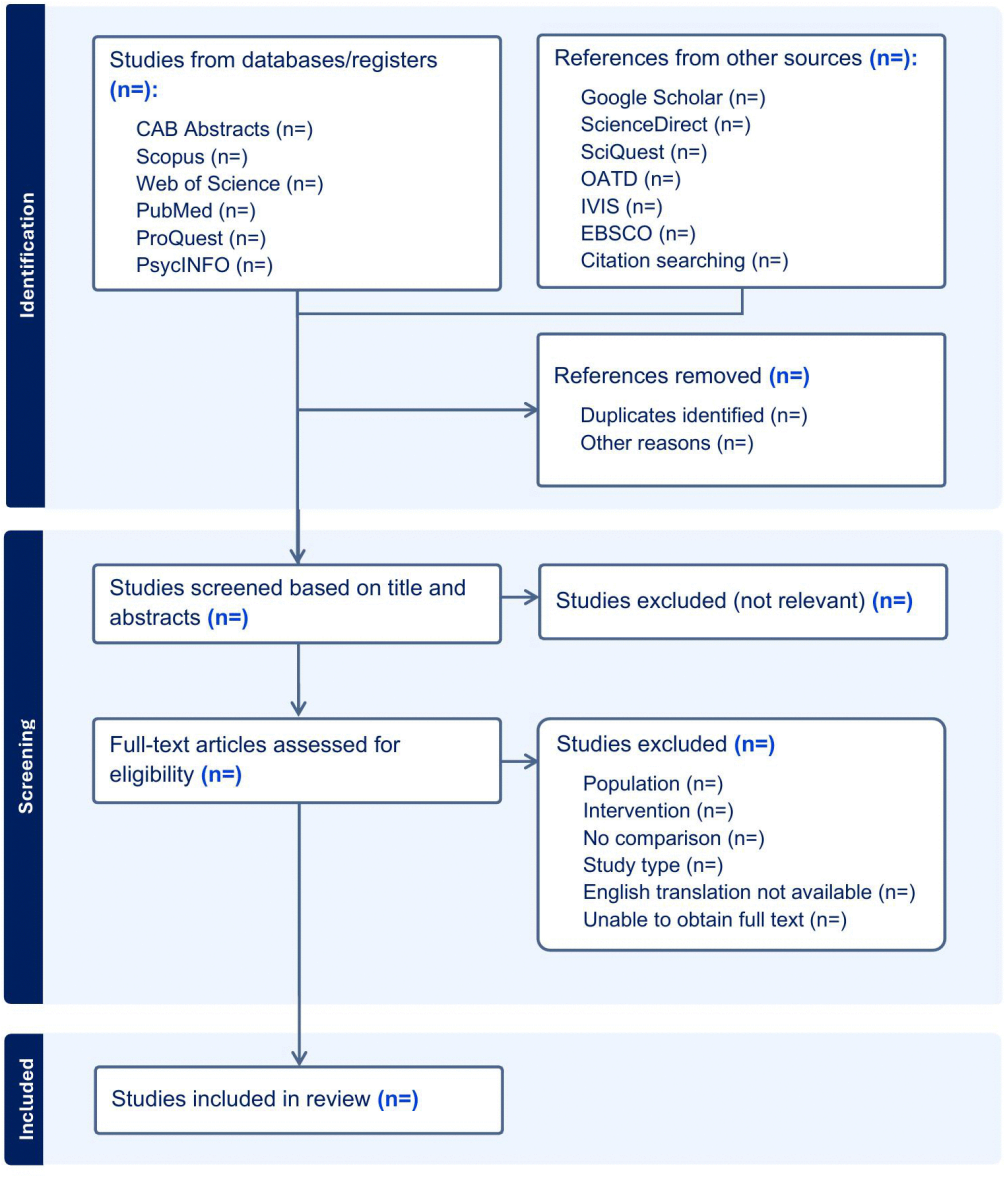
Flow diagram outlining the protocol review stages.

The selection process for the systematic review will be documented in a PRISMA flow diagram (Fig 1). Two independent reviewers will be involved in screening, data extraction, RoB, and confidence in evidence assessments. One reviewer (NC) will be involved in all stages of the review, while the second reviewer (a review team member) will vary depending on availability and potential conflicts of interest. Blinding of article selection will be facilitated using the Covidence software. Potentially relevant studies will be identified through title and abstract screening. Reviewers will not be blinded to the article’s bibliographic fields; therefore, bias will be mitigated through the use of two independent reviewers and predefined eligibility criteria. Each record will be assessed against the eligibility criteria within Covidence, and only records with a positive or unclear response to the inclusion criteria will proceed to full-text screening. Any disagreements will be resolved through discussion or arbitrated by a third, independent reviewer (HR). Reasons for exclusion of full-text articles will be recorded in Covidence at the first clear instance of ineligibility and will be documented in a “Characteristics of Excluded Studies” table.

#### Data collection process.

Two reviewers will independently extract data from the selected studies using a modified data extraction form within Covidence [63]. The form will be pilot tested on 5 articles to ensure all relevant data is being captured prior to full data extraction. Any changes to the extraction form will be updated and uploaded into OSF before extracting data from the remaining papers. Upon completion of data extraction, any discrepancies will be addressed through discussion and in consultation with a third independent reviewer (HR). While Covidence includes some machine learning that can extract certain types of data, all extracted data will be collected manually due to the complexity and variability in equine weaning research.

Authors of articles with insufficient information for data extraction will be contacted by email to attempt to clarify or obtain the required information. A follow-up email will be sent one month later, and authors will be given three months to provide the requested information. If requested information is not obtained and there is still insufficient information for data extraction, these articles will be excluded from data analysis and documented in the PRISMA flow diagram (Fig 1) and “Characteristics of Excluded Studies” table.

### Data items

#### Data extraction.

Data extracted from each article will include, but not be limited to, *article identification* (e.g., year of publication, country, language, funding, setting, contact author details), *study information and methods* (e.g., study aim, main objectives, study design, data collection methods, method of randomisation, sample sizes), *population* (e.g., age, sex, breed), *treatment interventions* (e.g., intervention name and description, intervention procedure, number of participants, conspecifics, weaning housing, environment, duration), and *primary outcomes* (e.g., outcome name and definition, unit of measure, scale, time points, statistical method, results).

#### Subgroups/Moderators.

Study-level characteristics will be analysed in predefined categories as subgroup factors in pairwise meta-analyses and as moderating factors in Bayesian NMA, and will include *age* (early (<5 months), average (5–7 months), late (> 7 months) [43]), *sex* (fillies, colts), *housing during weaning* (stable (<25m2), yard/barn (>25m2 to 0.2 ha), paddock (>0.2ha) [64]), and *conspecifics during weaning* (individual, pair, > 2 individuals). Additionally, outcome measures will be collected within predefined timeframes (Table 3), to help facilitate exploration of heterogeneity and identification of potential moderating factors.

**Table 3:** Pre- and post-weaning outcome timeframes categories.

Stage	Category	Definition
Pre-weaning	Pre-weaning	Within 7 days prior to weaning
Post-weaning	Immediate	Day 0 (from point of weaning and including the remainder of that day)
	Short-term	Days 1–2 post weaning
	Medium-term	Days 3–7 post weaning
	Longer-term	> 7 days post weaning

### Outcomes and prioritisation

Primary outcomes are those previously mentioned: physiological outcomes prioritising cortisol, heart rate, and body weight; and behavioural outcomes prioritising locomotion, vocalisation, agonistic behaviour, defecation, and eating. These outcomes will be prioritised for synthesis where multiple outcomes are reported. Where studies include multiple intervention arms or time points, double-counting will be avoided by combining groups or selecting the most relevant comparison. Each outcome type will be analysed separately, and where the same outcome is reported using different scales or units, established standardised effect measures will be used to improve comparability across studies. Outcomes reported in the results will be compared against those listed in their methods or protocols to identify selective reporting or missing outcome data. Trials with unresolved inconsistencies or serious methodological flaws will be excluded from the synthesis or included only narratively, with their limitations noted.

### Risk of bias

Sources of bias will be assessed alongside data extraction in Covidence using the Cochrane risk of bias tool (RoB v2) [65] for RCTs, and ROBINS-I (Risk of Bias in Non-randomised Studies – of Interventions) for non-RCTs [66]. The RoB v2 tool includes a judgement evaluation of bias from the randomisation process, deviations from the intended intervention, blinding, measurement of the outcome, missing outcome data and selective outcome reporting. In non-RCTs, biases include those associated with confounding, selection, intervention classification and deviations, missing data, outcome measurements, and reporting. The risk of bias due to missing evidence in the NMA (ROB-MEN) will examine potential within- and across-study biases [67]. All assessments of bias will be made independently by two reviewers using the criteria for each tool. Disagreements will be resolved via discussion or with a third reviewer (HR) serving as an arbitrator. The overall RoB assessment for each outcome will be used to assess confidence in the evidence and presented in a summary figure generated by Robvis (https://mcguinlu.shinyapps.io/robvis/) or R Shiny (https://cinema.ispm.unibe.ch/rob-men/).

### Data synthesis

Descriptive summaries of all included studies will be presented by intervention, study-level characteristics, and outcomes. Evidence tables and figures will be used to show qualitative and quantitative data as appropriate.

#### Effect measurements.

The primary effect measures for continuous outcomes will be the weighted mean difference (MD, 95% confidence interval (CI)) for outcomes measured on the same scale, and the standardised mean difference (SMD, 95% CI) for outcomes measured on different scales [68]. For dichotomous outcomes, the primary effect measure will be the risk ratio (RR) with 95% CI. Count data will be treated as continuous or dichotomous, depending on the outcome’s frequency. For multi-arm trials, correlations between effect estimates will be accounted for using arm-based models or variance-correction techniques to avoid unit-of-analysis errors.

Outcome measures will be collected for each intervention and study-level characteristic within predefined time frames, allowing assessment of immediate-, short- and longer-term effects [68]. When trials report multiple measurements of the same continuous outcome within a single time window, the effect estimates will be combined using inverse-variance weighting to produce a single summary value per time window, with the corresponding standard error (SE) calculated from the sum of the weights. For dichotomous outcomes, event counts and total observations from multiple measurements within each time window will be aggregated to derive a single study-level RR, 95% CI, and SE.

For pre- versus post-intervention comparisons, continuous outcomes will be calculated as the mean change (post-pre) when measured on the same scale, or as standardised mean change when measured on different scales, using the standard deviation (SD) of the change score [69]. For dichotomous outcomes, event counts and total observations will be used to calculate an RR comparing post- to pre-intervention periods. Where pre-post comparisons are not possible due to missing or incompatible baseline data, post-intervention endpoint values will be used to derive effect estimates (MD, SMD, or RR) for each time window. All effect estimates will be accompanied by their SE, serving as the summary statistics for both pairwise meta-analysis and Bayesian NMA [70].

When means and SD are not reported, the primary authors will be contacted to obtain the original data. If unavailable, means and SDs will be estimated from medians, ranges, or SE using established methods [69]. Graphically presented data will be digitally extracted. All assumptions and computations will be documented, their potential impact assessed through sensitivity analyses, and the resulting biases discussed.

#### Pairwise meta-analysis.

To assess the overall impact of artificial weaning on foal physiology and behaviour (objective one), pairwise random-effects meta-analysis will be used, as it is better able to account for variability between studies [69]. Analyses will be conducted using Cochrane’s RevMan (version 10.6.0; https://revman.cochrane.org) or R Statistical Software (version 4.6.0; www.r-project.org), or the version available at the time of analysis. Separate analyses will be conducted for each predefined time window. Research reporting only post-weaning scores will be excluded from SMD analyses to avoid mixing sources of variability.

Point estimates and 95% CIs will be presented in a Summary of Findings table, and conclusions will be based on consistency, direction, and magnitude of effects across studies rather than statistical P values alone [71]. Continuous outcome MDs will be reported in natural units, with positive values indicating an increase and negative values indicating a decrease in the outcome post-weaning. The relevance of these changes will depend on the nature and direction of each specific outcome. For outcomes measured using different scales, SMDs will be reported and interpreted according to Cohen’s rules (0.2 = small, 0.5 = moderate, 0.8 = large). For ease of interpretation, SMDs will, where possible, be re-expressed on a familiar scale. When both pre- and post-weaning continuous outcome values are positive and available, the ratio of means will be calculated to provide a relative measure of change. For dichotomous outcomes, both relative and absolute effects will be presented. Precise P values will be reported, but interpretation of their meaningfulness will be made in conjunction with 95% CIs and confidence in evidence ratings.

Statistical between-study heterogeneity will be assessed using the I² statistic and τ² estimates [69]. Data will be considered sufficiently homogeneous when I² is less than 50%, and τ² values are small. If data are not homogeneous, prediction intervals will be presented rather than the average intervention effects to account for the variability [72]. Sensitivity analyses comparing fixed-effect and random-effect estimates will be conducted to assess if small trials are affecting heterogeneity. Additional sources of heterogeneity will be explored using network meta-analysis [73].

#### Network meta-analysis.

The Bayesian NMA framework is well-suited for managing complex interventions, accounting for between-study heterogeneity, small sample sizes, sparse data, and uncertainty in effect estimates, and was therefore chosen as the method for this review [46]. A standard intervention-level NMA will be used to assess objective two, while a component-based NMA model will explore how moderating factors contribute to weaning outcomes in foals (objective three) [73]. Results from the NMA will inform the assessment and ranking of interventions and moderating factors, indicating which practices will improve foal welfare (objective four) [46].

#### Feasibility and network structure.

The NMA model will be used when three or more interventions can be linked through a connected evidence network and when outcomes are reported consistently across trials [46]. Studies will be eligible for inclusion if they compare at least two interventions and report sufficient aggregate-level data to calculate relative intervention effects. Connectivity will be visually assessed by mapping all available pairwise comparisons to produce a network diagram and characteristics tables [70]. Disconnected nodes will be excluded from the NMA and analysed separately using pairwise meta-analysis.

#### Model specification.

A Bayesian NMA will be conducted using a hierarchical random-effects framework using R Statistical Software [70]. The reference intervention was selected to provide a consistent benchmark against which all other interventions will be compared. Abrupt, individual weaning was chosen as it is a standard industry weaning practice [10,11]. Due to the likely small number of comparable studies and the absence of prior data to provide informative prior distributions, weakly informative priors will be used [70,74]. These priors are based on reasonable assumptions regarding plausible effect sizes and heterogeneity and will be used to improve model stability without strongly influencing the posterior estimates. For intervention and moderating factors, a Normal(0, 10) prior will be used, while the between-study standard deviation (τ) will be assigned a Half-Normal(0,1) prior, selected based on the likelihood of small trial sizes and sparse data sets.

Analyses will be performed using Markov Chain Monte Carlo (MCMC) simulations with three chains, each beginning with dispersed starting values to ensure thorough exploration of the posterior distributions [70]. A burn-in period of 10,000 iterations will be used to allow the chains to reach stationarity, followed by 50,000 sampling iterations to obtain posterior inferences. Both point estimates and 95% credible intervals will be reported. Convergence will be assessed using trace plots to visually inspect mixing and stability, and the Gelman-Rubin diagnostic, with values below 1.05 indicating convergence. If convergence is not satisfactory, the model will be re-run, increasing burn-in and sampling iterations by 10% to improve model stationarity until convergence is reached.

Ranking of interventions and moderating factors will be estimated using the Surface Under the Cumulative Ranking Curve (SUCRA) scores and mean ranks [46]. The results will be presented as a league table showing posterior mean effect sizes and 95% credible interval (CrI) for all pairwise comparisons, and rankograms will be generated to visualise the ranking probability of each intervention or study-level characteristic improving foal welfare (objective four).

#### Model fit.

The posterior mean residual deviance (D‾_*res*_) will be used to assess model fit. The D‾_*res*_ computed during MCMC iterations will be compared with the number of data points (the number of studies multiplied by the number of interventions). A D‾_*res*_ value close to the number of data points indicates a good fit, values substantially larger suggest a lack of fit, while smaller values indicate model overfit.

#### Sensitivity analyses.

Sensitivity analyses will be performed to examine the impact of bias and test the robustness of the results. Sensitivity analyses will include investigating the impact of missing data, the choice of effect measures and priors, and the exclusion of outlier, non-RCT, high RoB, and low confidence in evidence studies. Models will be compared with and without subgroups to assess the contribution of study-level characteristics to heterogeneity, and alternative categorisations of study-level definitions will also be tested.

#### Assumptions.

The validity of this model is based on three key assumptions – heterogeneity, transitivity, and coherence [46]. Heterogeneity between direct head-to-head comparisons will be examined using I² and ‾² as described in the pairwise meta-analysis section. When heterogeneity is present, component-based NMA and sensitivity analyses will help identify important effect modifiers [70].

Transitivity assumes that the population and distribution of effect modifiers are similar across comparisons [46]. The transitivity of the foal population is met through strict population inclusion criteria. Transitivity of key effect modifiers, including intervention characteristics and study conditions, will be assessed by examining whether these effect modifiers are similarly distributed across comparisons. Where substantial differences are identified, subgroup or sensitivity analyses will be conducted to explore the impact of these differences on the network, and comparisons will be excluded if transitivity assumptions cannot be met.

Coherence is the assumption that there is agreement between direct and indirect evidence [46]. Local coherence will be assessed using the node-splitting approach, separating indirect from direct evidence, to compare estimates for each intervention. The difference between these two estimates will be considered coherent when the 95% CrI includes zero. Additionally, if the calculated Bayesian P-values are not significant (>0.05) and the CrI overlaps, local coherence for that intervention arm is further supported. Global coherence across the network will be assessed by comparing the fit of a consistency model with an inconsistency model [70]. Model fit will be evaluated using DIC values. If the inconsistency model has lower DIC values (>3 points), global inconsistency will be inferred [69]. Residual deviance and leverage plots will be used to identify influential studies and those that may cause model misfit.

#### Synthesis without meta-analysis.

Network meta-analysis will not be used if the network diagram indicates insufficient connectivity, the assumptions of transitivity and consistency have not been met, or outcomes are too heterogeneous [46]. In this instance, where appropriate, results will be synthesised using pairwise random-effects meta-analysis. Where direct evidence is sparse or insufficiently homogeneous, and if pairwise meta-analysis is inappropriate, data will be synthesised using Synthesis Without Meta-analysis (SWiM) methods [75]. Results will be presented in tabulated or graphical form following the SWiM reporting guidelines [52]. The decision not to proceed with any meta-analyses will be documented and justified.

### Meta-bias

While it is acknowledged that publication or reporting bias can affect results, detecting such biases is often difficult [54]. Nevertheless, as it is standard practice to assess publication bias, contour-enhanced funnel plots will be created to display the publication spread and examine small-study effects visually. Any asymmetries will be cautiously interpreted in consultation with the sensitivity analyses results.

### Confidence in evidence

Confidence in the evidence will be assessed using the Grading of Recommendations, Assessment, Development and Evaluation (GRADE) framework for pairwise meta-analysis [76,77], and the Confidence in Network Meta-Analysis (CINeMA) for the NMA [78]. Two reviewers will independently assess the confidence in evidence for each identified outcome, categorised as ‘High’, ‘Moderate’, ‘Low’ or ‘Very low’, with a third reviewer serving as an arbitrator. Decisions regarding upgrades or downgrades in confidence will be documented, and the assessments will be presented in the summary of findings table. Whilst studies with low confidence in evidence will not be excluded from the review, sensitivity analyses will be used to explore the impact of lower-quality studies on the results, and interpretations will be weighted accordingly.

## Conclusion

This systematic review protocol will facilitate the exploration of artificial weaning practices, a process known to cause significant stress in domestic horses, to identify optimal strategies to enhance foal welfare. As public pressure to improve horse welfare continues to grow, the commencement of this systematic review will be timely. It will have the potential to make a significant contribution to enhancing foal welfare. The results of this review may inform breeders, stud managers, industry bodies, and policymakers about optimal comparative weaning practices based on current knowledge and highlight areas for future research.

### Amendments

All protocol amendments will be documented by NC and include the date, describe the change, and provide the rationale in a table of adjustments, which will be uploaded to OSF upon publication of the review. Since the initial registration of the review on OSF, the objectives and research questions have been expanded and refined to clarify the study’s aim. Due to the complexity of weaning interventions, network meta-analysis has been included to allow for indirect comparisons, the exploration of moderating factors, and the ranking of interventions, thereby enhancing the provision of recommendations. Additionally, the secondary outcomes outlined in the protocol registered on OSF have been redefined as study-level characteristics to reflect their true nature as subgroup and moderating factors, in keeping with PRISMA-P guidelines [48]. No additional protocol amendments beyond those already documented have been made.

## Supporting information

S1 Checklist PRISMA-P checklist. (PDF)
