# Supplementary material for: Determining optimal practices for foal weaning – A protocol for a systematic review and network meta-analysis
Source: PLoS One. 2026 Jul 1;21(7):e0352182. doi: 10.1371/journal.pone.0352182 (PMC13322514; doi:10.1371/journal.pone.0352182)
Supplement: S1 Checklist — (PDF) [file pone.0352182.s001.pdf]

PRISMA-P 2015[1] and relevant components of PRISMA-NMA[2] checklist
Completed on 21/11/2025 by N Cranston for:
Determining optimal practices for foal weaning based on physiological, behavioural, and welfare indices - A protocol for a systematic review and network meta-analysis

Section and topic	Item No	Checklist item (*text in italics specific to NMA*)	PRISMA-P	Location(s) in manuscript	PRISMA-NMA	Location(s) in manuscript
**ADMINISTRATIVE INFORMATION**						
Title:						
Identification	1a	Identify the report as a protocol of a systematic review *incorporating a network meta-analysis (or related form of meta-analysis).*	☑	Title page - Title	☑	Title page - Title
Update	1b	If the protocol is for an update of a previous systematic review, identify as such.		N/A		
Registration	2	If registered, provide the name of the registry (such as PROSPERO) and registration number.	☑	Abstract - Registration		
Authors:						
Contact	3a	Provide name, institutional affiliation, e-mail address of all protocol authors; provide physical mailing address of corresponding author.	☑	Title page - Authors		
Contributions	3b	Describe contributions of protocol authors and identify the guarantor of the review.	☑	Declarations - Authorship		
Amendments	4	If the protocol represents an amendment of a previously completed or published protocol, identify as such and list changes; otherwise, state plan for documenting important protocol amendments.	☑	Declarations - Amendments		
Support:						
Sources	5a	Indicate sources of financial or other support for the review.	☑	Declaration - Financial support		
Sponsor	5b	Provide name for the review funder and/or sponsor.	☑	Declaration - Financial support		
Role of sponsor	5c	Describe roles of funder(s), sponsor(s), and/or institution(s), if any, in developing the protocol.	☑	Declaration - Financial support		
**INTRODUCTION**						
Rationale	6	Describe the rationale for the review in the context of what is already known *including mention of why a network meta-analysis has been conducted.*	☑	Introduction	☑	Introduction - NMA paragraph 7
Objectives	7	Provide an explicit statement of the question(s) the review will address with reference to participants, interventions, comparators, and outcomes (PICO).	☑	Introduction - Objectives		

Section and topic	Item No	Checklist item (*text in italics specific to NMA)*	PRISMA-P	Location(s) Reported	PRISMA-NMA	Location(s) Reported
**METHODS**						
Eligibility criteria	8	Specify the study characteristics (such as PICO, study design, setting, time frame) and report characteristics (such as years considered, language, publication status) to be used as criteria for eligibility for the review. *Clearly describe eligible treatments included in the treatment network, and note whether any have been clustered or merged into the same node (with justification).*	☑	Methods - Eligibility criteria	☑	Methods - Eligibility criteria - Comparisons<br>Methods - Data items - Subgroups/Moderators
Information sources	9	Describe all intended information sources (such as electronic databases, contact with study authors, trial registers or other grey literature sources) with planned dates of coverage.	☑	Methods - Information sources		
Search strategy	10	Present draft of search strategy to be used for at least one electronic database, including planned limits, such that it could be repeated.	☑	Methods - Search strategy		
Study records:						
Data management	11a	Describe the mechanism(s) that will be used to manage records and data throughout the review.	☑	Methods - Study records - Data management		
Selection process	11b	State the process that will be used for selecting studies (such as two independent reviewers) through each phase of the review (that is, screening, eligibility and inclusion in meta-analysis).	☑	Methods - Study records - Selection process		
Data collection process	11c	Describe planned method of extracting data from reports (such as piloting forms, done independently, in duplicate), any processes for obtaining and confirming data from investigators.	☑	Methods - Study records - Data collection process		
Data items	12	List and define all variables for which data will be sought (such as PICO items, funding sources), any pre-planned data assumptions and simplifications.	☑	Methods - Data items - Data extraction<br>Methods - Data items - Subgroups/ Moderators		
Outcomes and prioritization	13	List and define all outcomes for which data will be sought, including prioritization of main and additional outcomes, with rationale	☑	Methods - Outcomes and prioritisation		

**Section and topic**	**Item No**	**Checklist item** (*text in italics specific to NMA*)	**PRISMA-P**	**Location(s) Reported**	**PRISMA-NMA**	**Location(s) Reported**
Risk of bias in individual studies	14	Describe anticipated methods for assessing risk of bias of individual studies, including whether this will be done at the outcome or study level, or both; state how this information will be used in data synthesis.	☑	Methods - Risk of bias		
Data synthesis	15a	Describe criteria under which study data will be quantitatively synthesised.	☑	Methods - Data synthesis - Synthesis without meta-analysis		
	15b	If data are appropriate for quantitative synthesis, describe planned summary measures, methods of handling data and methods of combining data from studies, including any planned exploration of consistency (such as I^2, Kendall's τ).	☑	Methods - Data synthesis - Effect measurements<br>Methods - Data synthesis - Pairwise meta-analysis<br>Methods - Data synthesis - Network meta-analysis		
	15c	Describe any proposed additional analyses (such as sensitivity or subgroup analyses, meta-regression, *alternative formulations of the treatment network; and use of alternative prior distributions for Bayesian analyses (if applicable).*	☑	Methods - Data synthesis - Pairwise meta-analysis<br>Methods - Data synthesis - Network meta-analysis - Model fit	☑	Methods - Data synthesis - Network meta-analysis - Sensitivity analysis
	15d	If quantitative synthesis is not appropriate, describe the type of summary planned.	☑	Methods - Data synthesis - Synthesis without meta-analysis		

Section and topic	Item No	Checklist item (*text in italics specific to NMA)*	PRISMA-P	Location(s) Reported	PRISMA-NMA	Location(s) Reported
NMA specific requirements	***S1***	Geometry of the network - Describe methods used to explore the geometry of the treatment network .		N/A	☑	Methods - Data synthesis - Network meta-analysis - Feasibility and network structure
	13	Summary measures - Describe the use of additional summary measures assessed, such as treatment rankings and surface under the cumulative ranking curve (SUCRA) values, as well as modified approaches used to present summary findings from meta-analyses.			☑	Methods - Data synthesis - Network meta-analysis - Model specification
	14	Planned methods of analysis including:<br>- Handling of multi-arm trials<br>- Selection of variance structure<br>- Selection of prior distributions in Bayesian analyses<br>- Assessment of model fit.			☑	Methods - Outcomes and prioritisation<br>Methods - Data synthesis - Network meta-analysis - Model specification<br>Methods - Data synthesis - Network meta-analysis - Model fit
	S2	Assessment of inconsistency - Describe the statistical methods used to evaluate the agreement of direct and indirect evidence in the treatment network(s) studied. Describe efforts taken to address its presence when found.			☑	Methods - Data synthesis - Network meta-analysis - Assumptions
Meta-bias(es)	16	Specify any planned assessment of meta-bias(es) (such as publication bias across studies, selective reporting within studies).	☑	Methods - Meta-bias		
Confidence in cumulative evidence	17	Describe how the strength of the body of evidence will be assessed (such as GRADE).	☑	Methods - Confidence in evidence		

[1]Moher D, Shamseer L, Clarke M, Ghersi D, Liberati A, Petticrew M, Shekelle P and Stewart LA (2015) Preferred reporting items for systematic review and meta-analysis protocols (PRISMA-P) 2015 statement. Systematic Reviews 4, 1. doi:10.1186/2046-4053-4-1

[2]Hutton B, Salanti G, Caldwell DM, Chaimani A, Schmid CH, Cameron C, Ioannidis JP, Straus S, Thorlund K, Jansen JP, Mulrow C, Catalá-López F, Gøtzsche PC, Dickersin K, Boutron I, Altman DG and Moher D (2015) The PRISMA extension statement for reporting of systematic reviews incorporating network meta-analyses of health care interventions: checklist and explanations. Annals of Internal Medicine 162, 777-84. doi:10.7326/m14-2385
