# Supplementary material for: Determining optimal practices for foal weaning – A protocol for a systematic review and network meta-analysis
Source: PLoS One. 2026 Jul 1;21(7):e0352182. doi: 10.1371/journal.pone.0352182 (PMC13322514; doi:10.1371/journal.pone.0352182)
Supplement: Attachment — Submitted filename: Scientific Comments on the Review.docx [file pone.0352182.s002.docx]

**Scientific Comments on the Review (Point-by-Point Evaluation and Suggested Corrections)**

**1. Line 77–84: Introduction – Contextualizing Equine Welfare Concerns**

*Comment:*
The opening effectively establishes the societal and ethical context for improving equine welfare, citing relevant literature (e.g., Douglas et al., 2022; Heleski, 2023). However, the claim that "the horse industry faces increasing public pressure" would benefit from more specific data or survey-based evidence to substantiate the trend. Consider citing public opinion studies or reports from animal welfare NGOs (e.g., World Horse Welfare) to strengthen this assertion.

*Suggested revision:*

"Growing public scrutiny of equine welfare, evidenced by increased media coverage and consumer surveys (e.g., World Horse Welfare, 2023), has placed pressure on the industry..."

**2. Line 79–81: Reference to 2021 Olympic Incident**

*Comment:*
While the reference to the 2021 Olympic Pentathlon incident is accurate and impactful, the citation (Cuckson, 2021) appears to refer to a news report rather than a peer-reviewed source. For scientific rigor in a systematic review protocol, it is preferable to cite formal investigations or official reports (e.g., FEI inquiry outcomes) where available.

*Suggested revision:*
Replace with:

"...such as the 2021 Olympic Modern Pentathlon horse incident (FEI, 2021), which prompted widespread criticism and calls for reform."

**3. Line 86–89: Broadening Scope to Weaning Practices**

*Comment:*
This transition is logical and well-supported. The inclusion of Pearson and Douglas (2025) strengthens the argument that weaning falls within broader welfare concerns. However, ensure that this reference is not forward-dated relative to the current date (April 2026); if unpublished or in press, clarify its status appropriately.

**4. Line 91–104: Definition and Stress of Weaning**

*Comment:*
The definition of weaning as “nutritional separation” aligns with Latham and Mason (2008) and Henry et al. (2020), but could be expanded slightly to acknowledge behavioural and emotional components of dam-offspring bonding, which contribute to stress responses. This would enhance biological plausibility.

*Suggested addition:*

"Weaning involves not only nutritional separation but also disruption of a strong social bond, contributing to significant psychological stress in foals."

Also, consider clarifying that abrupt weaning often includes abrupt *social* separation, which compounds stress beyond nutrition alone.

**5. Line 107–117: Heterogeneity in Gradual Weaning Definitions**

*Comment:*
This is a critical and well-articulated point. The lack of standardised definitions for "gradual weaning" across studies significantly undermines comparability. To strengthen this section, explicitly define how your review will categorise gradual vs. abrupt weaning based on operational criteria (e.g., duration of separation phases, frequency of contact).

*Suggested clarification in Methods:*

Add under *Interventions*:

"Gradual weaning will be operationally defined as any protocol involving staged physical separation over ≥3 days, including partial-contact systems (e.g., fenceline weaning), while abrupt weaning refers to immediate and complete separation without prior acclimatisation."

**6. Line 119–125: Additional Management Strategies**

*Comment:*
The list of strategies (nannies, diet modification, later weaning, pharmacology) is comprehensive. However, grouping pharmacological interventions (e.g., anxiolytics) with management practices may conflate fundamentally different approaches—one modifies physiology, others modify environment. Consider distinguishing between **management-based** and **medical/pharmacological** interventions in analysis.

*Suggestion:*
In Data Items or Subgroups, classify interventions into categories:

- Environmental (housing, conspecifics)
- Temporal (age at weaning, weaning method)
- Dietary (pre-/post-weaning nutrition)
- Medical (pharmaceutical use)

This improves interpretability in NMA.

**7. Line 130–140: Long-Term Consequences of Weaning Stress**

*Comment:*
The paragraph convincingly links early-life stress to long-term developmental impacts. However, the citation of Valenchon et al. (2025) again raises concern about temporal validity (assuming current date is April 2026). If this work has not yet been published, it should be cited as "in press" or removed until verified.

Additionally, consider adding a sentence linking these long-term effects directly to economic and performance implications for breeders, enhancing translational relevance.

**8. Line 156–165: Justification for Systematic Review and NMA**

*Comment:*
Strong justification for using both pairwise and network meta-analysis. However, note that Bayesian NMA requires careful handling of multi-arm trials and assumptions about independence. Clarify whether multi-arm studies will be accounted for using appropriate methods (e.g., arm-based or contrast-based models with covariance adjustment).

*Suggested addition in Methods:*

"For multi-arm trials, correlations between effect estimates will be accounted for using arm-based models or variance-correction techniques to avoid unit-of-analysis errors."

**9. Line 219–222: Timeline of Review Process**

*Comment:*
Given that full-text screening is expected by July 2026 and submission by late 2027, the statement appears plausible. However, since the initial search was conducted in August 2025, confirm that a **study update search** will be performed immediately before final synthesis (as noted in line 339), particularly given the two-year gap.

Ensure this is clearly stated to maintain currency.

**10. Line 240–261: Intervention Criteria**

*Comment:*
Clear distinction between planned and unplanned weaning is appropriate. However, the exclusion of wild/feral populations is justified but could be nuanced—some feral studies (e.g., on mustangs or brumbies) provide valuable baseline data on natural weaning patterns. While excluded from quantitative synthesis, consider mentioning their value in supplementary discussion.
