# Supplementary material for: Determining optimal practices for foal weaning – A protocol for a systematic review and network meta-analysis
Source: PLoS One. 2026 Jul 1;21(7):e0352182. doi: 10.1371/journal.pone.0352182 (PMC13322514; doi:10.1371/journal.pone.0352182)
Supplement: Attachment — Submitted filename: Response_to_Reviewers.docx [file pone.0352182.s003.docx]

**PLoS One Reviewer Comments**

- Sections in italics are sections from the manuscript
- Sections in red writing indicate where changes have been made
- Line numbers are in reference to the Manuscript_Revised.doc

| **Reviewer** | **Comment** | **Action** |
| --- | --- | --- |
| Journal | 1. Please ensure that your manuscript meets PLOS ONE's style requirements, including those for file naming. The PLOS ONE style templates can be found at   [https://journals.plos.org/plosone/s/file?id=wjVg/PLOSOne_formatting_sample_main_body.pdf](https://aus01.safelinks.protection.outlook.com/?url=https%3A%2F%2Ftrack.editorialmanager.com%2FCL0%2Fhttps%3A%252F%252Fjournals.plos.org%252Fplosone%252Fs%252Ffile%253Fid%3DwjVg%252FPLOSOne_formatting_sample_main_body.pdf%2F1%2F010f019d89c897a5-60f94f9e-219d-4b3c-8c46-d350ee1a368d-000000%2F4BPGEse9D9Kd4D4SGUIFZG7vz-DJmxy77JqtaadAhqY%3D258&data=05%7C02%7Cncranston%40csu.edu.au%7Cc260ba50b8f1406d35d108de99cc24db%7Cf0f76207a6104fc0b4a35d797fe5283c%7C0%7C0%7C639117299664667219%7CUnknown%7CTWFpbGZsb3d8eyJFbXB0eU1hcGkiOnRydWUsIlYiOiIwLjAuMDAwMCIsIlAiOiJXaW4zMiIsIkFOIjoiTWFpbCIsIldUIjoyfQ%3D%3D%7C0%7C%7C%7C&sdata=jXFkVxuLVMueZRP%2BgrESC70cHw7G0d5LTMnVxwmZ8pg%3D&reserved=0) and  [https://journals.plos.org/plosone/s/file?id=ba62/PLOSOne_formatting_sample_title_authors_affiliations.pdf](https://aus01.safelinks.protection.outlook.com/?url=https%3A%2F%2Ftrack.editorialmanager.com%2FCL0%2Fhttps%3A%252F%252Fjournals.plos.org%252Fplosone%252Fs%252Ffile%253Fid%3Dba62%252FPLOSOne_formatting_sample_title_authors_affiliations.pdf%2F1%2F010f019d89c897a5-60f94f9e-219d-4b3c-8c46-d350ee1a368d-000000%2FBCsdfY99EkDS_MCmsYFLopwmsRfO5Bcd-KHy_gZKpXI%3D258&data=05%7C02%7Cncranston%40csu.edu.au%7Cc260ba50b8f1406d35d108de99cc24db%7Cf0f76207a6104fc0b4a35d797fe5283c%7C0%7C0%7C639117299664684991%7CUnknown%7CTWFpbGZsb3d8eyJFbXB0eU1hcGkiOnRydWUsIlYiOiIwLjAuMDAwMCIsIlAiOiJXaW4zMiIsIkFOIjoiTWFpbCIsIldUIjoyfQ%3D%3D%7C0%7C%7C%7C&sdata=abuFkzxlpDsWDb16GfsNpXsA8XsGsiKJ6F2gC0GFoUw%3D&reserved=0) | The manuscript has been reformatted as per PLOS One style requirements, in accordance with the attached guidelines. |
| Journal | 2. We note that the grant information you provided in the ‘Funding Information’ and ‘Financial Disclosure’ sections do not match.  When you resubmit, please ensure that you provide the correct grant numbers for the awards you received for your study in the ‘Funding Information’ section. | Thank you, we have added an updated version to the cover letter as requested. |
| Journal | 3. Please note that funding information should not appear in any section or other areas of your manuscript. We will only publish funding information present in the Funding Statement section of the online submission form. Please remove any funding-related text from the manuscript. | Funding related information has been removed from the manuscript and will only be reported within the Funding Statement section on submission. |
| Journal | 4. Thank you for stating the following in the Competing Interests section:  “I have read the journal's policy and the authors of this manuscript have the following competing interests:  JL is a registered equine breeder with a financial stake in the industry. The remaining authors declare no financial competing interests.  There is a possibility that one or more members of this review team will be co-authors of papers under review. In such instances, the author will not participate in the screening, data extraction, quality assessment, or synthesis of that paper to avoid any conflict of interest.”  Please confirm that this does not alter your adherence to all PLOS ONE policies on sharing data and materials, by including the following statement: "This does not alter our adherence to  PLOS ONE policies on sharing data and materials.” (as detailed online in our guide for authors  <http://journals.plos.org/plosone/s/competing-interests>).  If there are restrictions on sharing of data and/or materials, please state these. Please note that we cannot proceed with consideration of your article until this information has been declared.  Please include your updated Competing Interests statement in your cover letter; we will change the online submission form on your behalf. | The cover letter has been updated to reflect the competing interests statement, with the requested appropriate acknowledgement. |
| Journal | 5. PLOS requires an ORCID iD for the corresponding author in Editorial Manager on papers submitted after December 6th, 2016. Please ensure that you have an ORCID iD and that it is validated in Editorial Manager. To do this, go to ‘Update my Information’ (in the upper left-hand corner of the main menu), and click on the Fetch/Validate link next to the ORCID field. This will take you to the ORCID site and allow you to create a new iD or authenticate a pre-existing iD in Editorial Manager | ORCID link validated in NC's profile |
| Journal | 6. We note you have included a table to which you do not refer in the text of your manuscript. Please ensure that you refer to Table 1 in your text; if accepted, production will need this reference to link the reader to the Table. | The references to all tables have been checked to ensure they are cited. |
| Journal | 7. If the reviewer comments include a recommendation to cite specific previously published works, please review and evaluate these publications to determine whether they are relevant and should be cited. There is no requirement to cite these works unless the editor has indicated otherwise. | Suggested references were reviewed for relevance, and appropriate citations have been incorporated where relevant. |
| Reviewer 1 | 1. Line 77–84: Introduction – Contextualizing Equine Welfare Concerns  Comment:  The opening effectively establishes the societal and ethical context for improving equine welfare, citing relevant literature (e.g., Douglas et al., 2022; Heleski, 2023).  However, the claim that "the horse industry faces increasing public pressure" would benefit from more specific data or survey-based evidence to substantiate the trend. Consider citing public opinion studies or reports from animal welfare NGOs (e.g., World Horse Welfare) to strengthen this assertion.  Suggested revision:  "Growing public scrutiny of equine welfare, evidenced by increased media coverage and consumer surveys (e.g., World Horse Welfare, 2023), has placed pressure on the industry..." | Thank you for the suggestion. We have incorporated the suggestion, including additional public survey references.  *(Line 49)*  The horse industry faces increasing public pressure to improve equine welfare [1, 2]. *The growing public scrutiny of equine welfare, evidenced by increased media coverage and consumer surveys [3, 4] have placed pressure on the industry to improve the ethical treatment of horses (Equus caballus) [2].*  *[3] Thoroughbred Welfare Initiative, 2021*  *[4] Equine Ethics and Wellbeing Commission (FEI), 2022)* |
| Reviewer 1 | 2. Line 79–81: Reference to 2021 Olympic Incident  Comment:  While the reference to the 2021 Olympic Pentathlon incident is accurate and impactful, the citation (Cuckson, 2021) appears to refer to a news report rather than a peer-reviewed source. For scientific rigor in a systematic review protocol, it is preferable to cite formal investigations or official reports (e.g., FEI inquiry outcomes) where available.  Suggested revision:  Replace with:  "...such as the 2021 Olympic Modern Pentathlon horse incident (FEI, 2021), which prompted widespread criticism and calls for reform." | We believe the previously suggested revision has addressed this concern. |
| Reviewer 1 | 3. Line 86–89: Broadening Scope to Weaning Practices  Comment:  This transition is logical and well-supported. The inclusion of Pearson and Douglas (2025) strengthens the argument that weaning falls within broader welfare concerns.  However, ensure that this reference is not forward-dated relative to the current date (April 2026); if unpublished or in press, clarify its status appropriately. | This reference is published in the Equine Veterinary Journal and therefore no change was required.  *(Line 668)*  *Pearson G, Douglas J. Start ‘em young, treat ‘em right: How horses’ early life experiences can set them up for success in life. Equine Veterinary Journal. 2025;57(3):540-5. doi: 10.1111/evj.14485.* |
| Reviewer 1 | 4. Line 91–104: Definition and Stress of Weaning  Comment:  The definition of weaning as “nutritional separation” aligns with Latham and Mason (2008) and Henry et al. (2020), but could be expanded slightly to acknowledge behavioural and emotional components of dam-offspring bonding, which contribute to stress responses. This would enhance biological plausibility.  Suggested addition:  "Weaning involves not only nutritional separation but also disruption of a strong social bond, contributing to significant psychological stress in foals."  Also, consider clarifying that abrupt weaning often includes abrupt social separation, which compounds stress beyond nutrition alone. | The original text was retained to maintain the accuracy of the definition of the term weaning, with the suggested additions incorporated later in the paragraph.  *(Change at line 69)*  *Weaning is recognised as one of the most stressful events in a horse's life [9-12]. As with other mammals, weaning is defined as the nutritional separation from the mare [11, 13]. In naturalistic settings, weaning usually occurs spontaneously around nine months of age, with foals remaining with their maternal herd until they are two to three years old [11]. In domestic settings, however, weaning is usually artificially implemented and generally occurs between four and eight months of age [9-11, 14, 15]. In addition to nutritional separation, weaning also involves complete physical, visual, and auditory separation of the foal from the mare. The splitting of the mare and foal dyad is defined as either abrupt weaning, due to immediate separation, or gradual weaning if separation occurs in stages. Weaning may also occur in an unfamiliar environment and often involves individual foals kept in complete isolation.* *Domestic weaning therefore involves not only nutritional separation but also the disruption of strong social bonds, contributing to significant psychological stress in foals, which is further compounded in those weaned abruptly.* |
| Reviewer 1 | 5. Line 107–117: Heterogeneity in Gradual Weaning Definitions  Comment:  This is a critical and well-articulated point. The lack of standardised definitions for "gradual weaning" across studies significantly undermines comparability. To strengthen this section, explicitly define how your review will categorise gradual vs. abrupt weaning based on operational criteria (e.g., duration of separation phases, frequency of contact).  Suggested clarification in Methods:  Add under Interventions:  "Gradual weaning will be operationally defined as any protocol involving staged physical separation over ≥3 days, including partial-contact systems (e.g., fenceline weaning), while abrupt weaning refers to immediate and complete separation without prior acclimatisation." | Thank you for the feedback. The text has been revised to reflect the reviewers' suggestion.  *(Methods/Intervention section - Line 188)*  *Abrupt weaning will be characterised as the immediate and complete separation of the foal from the dam without prior acclimatisation. This may occur individually (reference intervention), in pairs or in groups of foals, but without the presence of other adults. The reference intervention will be compared to alternative weaning interventions, including:*  *1) Abrupt weaning with a nanny (adult horse): foal weaned abruptly from the dam but left in the care of another adult horse;*  *2) Progressive removal of mares: progressive removal of 1-2 dams from an established dam-foal herd through the abrupt weaning of the foal over ≥ 2 days until all mares have been removed;*  *3) Gradual acclimatisation: foals gradually acclimatised to separations prior to (≥ 2 weeks) the commencement of the weaning process;*  *4) Gradual partial contact: partial contact maintained for ≥ 2 days prior to complete separation (e.g. fenceline, nutritional separation via covering udder);*  *5) Gradual over time: increasing separation time ≥ 2 days prior to complete separation;*  *6) Gradual over distance: increasing the distance separated over ≥ 2 days prior to complete separation; and*  *7) Other: any other weaning intervention not covered by previous definitions.* |
| Reviewer 1 | 6. Line 119–125: Additional Management Strategies  Comment:  The list of strategies (nannies, diet modification, later weaning, pharmacology) is comprehensive. However, grouping pharmacological interventions (e.g., anxiolytics) with management practices may conflate fundamentally different approaches—one modifies physiology, others modify environment. Consider distinguishing between management-based and medical/pharmacological interventions in analysis.  Suggestion:  In Data Items or Subgroups, classify interventions into categories:  • Environmental (housing, conspecifics)  • Temporal (age at weaning, weaning method)  • Dietary (pre-/post-weaning nutrition)  • Medical (pharmaceutical use)  This improves interpretability in NMA. | Clarification on the definitions of "management intervention" has been provided in the Methods to distinguish between factors treated as study interventions versus those analysed as subgroup/moderating variables. In addition, management-based interventions have been categorised in line with the reviewers' suggestion .  *(Methods/Interventions - Line 208)*  *Any additional management strategy specifically explored at the study treatment level, while keeping the primary weaning intervention constant, will be classified as a management intervention. The management interventions will be categorised as:*  *• Environmental (housing, conspecifics, handling)*  *• Temporal (age at weaning)*  *• Dietary (pre/post-weaning nutrition)*  *• Medical (pharmaceutical use).*  *However, when these factors are observed but not evaluated as a treatment-level comparison, they will be considered study-level characteristics and explored as subgroup or moderating factors. Additionally, experiments reporting temporary separations of the target population from their dam, with a management intervention aimed at reducing stress during the separation will also be included.* |
| Reviewer 1 | 7. Line 130–140: Long-Term Consequences of Weaning Stress  Comment:  The paragraph convincingly links early-life stress to long-term developmental impacts. However, the citation of Valenchon et al. (2025) again raises concern about temporal validity (assuming current date is April 2026). If this work has not yet been published, it should be cited as "in press" or removed until verified. | This reference has now been published in *Nature Communications*, and the reference has therefore been updated accordingly.  *(Line 747)*  *Valenchon M, Reigner F, Lefort G, Adriaensen H, Gesbert A, Barrière P, et al. Affiliative behaviours regulate allostasis development and shape biobehavioural trajectories in horses. Nature Communications. 2026;17(1):47. doi: 10.1038/s41467-025-66729-1.* |
| Reviewer 1 | Additionally, consider adding a sentence linking these long-term effects directly to economic and performance implications for breeders, enhancing translational relevance. | Thank you for the suggestion, a linking sentence has been added.  *(Line 97)*  *This, in turn, can affect learning ability, fearfulness, and handling efficiency which influence performance, and ultimately the commercial value of the horse [37-41]. Therefore, it is in the industry’s best interest to adopt weaning practices that safeguard foal welfare.* |
| Reviewer 1 | 8. Line 156–165: Justification for Systematic Review and NMA  Comment:  Strong justification for using both pairwise and network meta-analysis. However, note that Bayesian NMA requires careful handling of multi-arm trials and assumptions about independence. Clarify whether multi-arm studies will be accounted for using appropriate methods (e.g., arm-based or contrast-based models with covariance adjustment).  Suggested addition in Methods:  "For multi-arm trials, correlations between effect estimates will be accounted for using arm-based models or variance-correction techniques to avoid unit-of-analysis errors." | Great feedback, thank you. The text has been revised to reflect the reviewer's suggestion.  *(Methods/Effect measurements - Line 436)*  *For multi-arm trials, correlations between effect estimates will be accounted for using arm-based models or variance-correction techniques to avoid unit-of-analysis errors.* |
| Reviewer 1 | 9. Line 219–222: Timeline of Review Process  Comment:  Given that full-text screening is expected by July 2026 and submission by late 2027, the statement appears plausible. However, since the initial search was conducted in August 2025, confirm that a study update search will be performed immediately before final synthesis (as noted in line 339), particularly given the two-year gap.  Ensure this is clearly stated to maintain currency. | Thank you, the text has been updated. The protocol now specifies that the search will be repeated immediately prior to the data synthesis stage to capture any new published studies.  *(Line 296)*  *The search will be repeated immediately prior to data synthesis to identify any new research and again if the review is not published within 12 months of the previous search. Any studies published after this time will be noted but excluded from the synthesis.* |
| Reviewer 1 | 10. Line 240–261: Intervention Criteria  Comment:  Clear distinction between planned and unplanned weaning is appropriate. However, the exclusion of wild/feral populations is justified but could be nuanced—some feral studies (e.g., on mustangs or brumbies) provide valuable baseline data on natural weaning patterns. While excluded from quantitative synthesis, consider mentioning their value in supplementary discussion. | Thank you for this suggestion. We plan to acknowledge the relevance of feral/wild population studies in the systematic review discussion. |
| Reviewer 2 | The inclusion of diverse study designs (e.g., observational and case series) may introduce heterogeneity and bias; further justification and clarification of their role in synthesis are needed | Thank you, the text has been updated based on the comments. The role and justification for inclusion of the diverse study designs have been included.  *(Line 250)*  *Inclusion of these study designs will ensure a comprehensive assessment of the available evidence where high-quality comparative studies are limited. Narrative synthesis without meta-analysis will only be undertaken if higher-quality evidence is insufficient [52].* |
| Reviewer 2 | The use of the Cochrane RoB v1 tool should be justified or updated to RoB 2. | The risk of bias assessment has been updated to use the Cochrane RoB v2 tool. Thank you for this suggestion.  *(Line 412)*  *Sources of bias will be assessed alongside data extraction in Covidence using the Cochrane risk of bias tool (RoB v2) [65] for RCTs, and ROBINS-I (Risk of Bias in Non-randomised Studies - of Interventions) for non-RCTs [66].* |
| Reviewer 2 | Outcome heterogeneity requires clearer strategies for harmonisation and prioritisation. | We have clarified the strategies for outcome harmonisation and prioritisation in the Methods/Outcomes and prioritisation section.  *(Line 398)*  *Primary outcomes are those previously mentioned: physiological outcomes prioritising cortisol, heart rate, and body weight; and behavioural outcomes prioritising locomotion, vocalisation, agonistic behaviour, defecation, and eating. These outcomes will be prioritised for synthesis where multiple outcomes are reported. Where studies include multiple intervention arms or time points, double-counting will be avoided by combining groups or selecting the most relevant comparison. Each outcome type will be analysed separately, and where the same outcome is reported using different scales or units, established standardised effect measures will be used to improve comparability across studies. Outcomes reported in the results will be compared against those listed in their methods or protocols to identify selective reporting or missing outcome data. Trials with unresolved inconsistencies or serious methodological flaws will be excluded from the synthesis or included only narratively, with their limitations noted.* |
| Reviewer 2 | The assumptions underlying NMA, particularly transitivity, need stronger justification and explanation of how violations will be handled. | Thank you for the comment, justification has been added. As per the reviewers' comments, the assumptions underlying the NMA, particularly transitivity have been justified with explanations as to how violations will be handled included.  *(Line 567)*  *Transitivity of key effect modifiers, including intervention characteristics and study conditions, will be assessed by examining whether these effect modifiers are similarly distributed across comparisons. Where substantial differences are identified, subgroup or sensitivity analyses will be conducted to explore the impact of these differences on the network, and comparisons will be excluded if transitivity assumptions cannot be met.* |
| Reviewer 2 | Additionally, the choice of Bayesian priors should be justified and supported with references. | Agreed. The choice of Bayesian priors has been clarified and further justified in the Methods.  *(Line 518)*  *Due to the likely small number of comparable studies and the absence of prior data to provide informative prior distributions, weakly informative priors will be used [70, 74]. These priors are based on reasonable assumptions regarding plausible effect sizes and heterogeneity and will be used to improve model stability without strongly influencing the posterior estimates.* |
| Reviewer 2 | The Introduction could be more concise, and tense should consistently reflect a protocol. | Minor edits were made to improve conciseness of the Introduction, while retaining necessary background and context for a systematic review protocol. Additionally, we have revised the manuscript to ensure tense consistently reflects a prospective protocol, with future‑oriented wording used for planned activities and past tense retained only where actions have already been completed. |
| Reviewer 2 | The Google Scholar search limit requires justification. | A justification for the Google Scholar search limit has been added in line with established methodological guidance.  *(Line 291)*  *… and the first 200 references (sorted by relevance) identified in Google Scholar will be included, as relevance declines rapidly, and this approach is recommended to balance sensitivity with feasibility [53, 55].* |
| Reviewer 2 | Consider reporting inter-rater reliability, clarifying subgroup definitions, and acknowledging limitations related to publication bias and language restrictions. | We have acknowledged limitations related to publication bias and language of the search as suggested.  *(Line 294)*  *Despite these measures, some degree of publication bias may remain, and conducting the search in English may limit the retrieval of relevant studies published in other languages.*  Additionally, we have re-checked the screening process and can confirm it adheres to current systematic review practices, including independent dual review and consensus‑based resolution of disagreements, with third‑party independent resolution where required. We also reviewed the subgroup definitions provided in the Methods (age at weaning, housing environment, and conspecific exposure) and confirmed that explicit category definitions and reference‑based thresholds are clearly described (see Section starting at line 385) |
| Reviewer 3 | 1.Title: Shorten the title for readability; | Shortened to:  *Determining optimal practices for foal weaning - a protocol for a systematic review and network meta-analysis* |
| Reviewer 3 | 2. Abstract: Clarify the type of study (protocol only) and explicitly state that no empirical results are included. | The identification of the study being a protocol only and statement that the paper contains no empirical evidenced added to the end of conclusion section of the abstract. Also updated the introduction to explicitly state this is a systematic review protocol.  *(Line 43)*  *This paper outlines the protocol that will be used and does not contain any empirical results.*  *(Line 115)*  *This systematic review protocol is proposed to critically evaluate the available literature on foal weaning.* |
| Reviewer 3 | 3.Confirm the OSF registration DOI (<https://doi.org/10.17605/OSF.IO/DJWQY>) is active and publicly accessible. Add a sentence confirming no protocol amendments beyond those already documented. | We have verified that the OSF DOI registration is active and publicly accessible. The requested statement confirming no additional protocol amendments have been included at the end of the amendments section.  *(Line 638)*  *No additional protocol amendments beyond those already documented have been made.* |
| Reviewer 3 | 4.Standardize terminology: abrupt weaning and gradual weaning definitions are clear but should be formally defined in a single box or paragraph for consistency. | The definitions of abrupt and gradual weaning have been revised to be more explicit to improve clarity and consistency. Thank you for this suggestion.  *(Line 66)*  *In addition to nutritional separation, weaning also involves complete physical, visual, and auditory separation of the foal from the mare. The splitting of the mare and foal dyad is defined as either abrupt weaning, due to immediate separation, or gradual weaning if separation occurs in stages.* |
| Reviewer 3 | 5.Explicitly state whether language is restricted to English (currently implied but not clearly stated). | Done, thank you. Language is not restricted at the search stage, so this has been made explicit in the eligibility criteria.  *(Line 258)*  *There will be no limitations based on the year of publication, geographical location, publication type, or language. Articles not written in English will be translated where possible, and those that cannot be translated will be excluded from full-text screening.* |
| Reviewer 3 | 6. Methods: Specify the exact six databases used (listed in Table 1 but not fully listed in the main text);  In NMA: Clearly state the reference intervention (abrupt individual weaning) earlier in Methods;  Define time-frame categories (immediate/short/medium/long-term post-weaning) in a table for quick reference. | We have reviewed this section and can confirm that the six bibliographic databases searched are listed in the main text (Information Sources section; paragraph starting at line 280).  While the reference intervention (abrupt individual weaning) is specified within the network meta-analysis model specification, where it is directly relevant to the analytical framework, we have also included this earlier in the intervention section as requested.  *(Line188)*  *Abrupt weaning will be characterised as the immediate and complete separation of the foal from the dam without prior acclimatisation. This may occur individually (reference intervention), in pairs or in groups of foals, but without the presence of other adults. The reference intervention will be compared to alternative weaning interventions, including:*  A table defining the pre- and post-weaning timeframe categories has also been added to enable quick reference (Table 3 - Line 395). |
| Reviewer 3 | 1.Add a brief graphical abstract or flow diagram of review stages for readability. | A PRISMA flow diagram has been added to the manuscript to visually summarise the review process and improve readability. Thank you for the suggestion.  *(Line 340 and Fig 1 attached document)*  *The selection process for the systematic review will be documented in a PRISMA flow diagram (Fig 1).* |
| Reviewer 3 | 2.Include a list of predefined primary/secondary outcomes in a table. | A table listing the primary and secondary outcomes has been included as requested (Table 1 - Line 244) |
| Reviewer 3 | 3.Specify planned software versions (R, RevMan, Covidence) if known. | Thank you for the suggestion. Software details for RevMan and R have been updated to reflect the versions available at the time of manuscript preparation. Covidence has been checked and does not provide a fixed version numbering system.  (Line 465)  *To assess the overall impact of artificial weaning on foal physiology and behaviour (objective one), pairwise random-effects meta-analysis will be used, as it is better able to account for variability between studies [69]. Analyses will be conducted using Cochrane's RevMan (version 10.6.0; https://revman.cochrane.org) or R Statistical Software (version 4.6.0; www.r-project.org), or the version available at the time of analysis.* |
| Reviewer 4 | No comments |  |
| Additional | Added PRISMA-P/NMA checklist as supplement | *(Line166 & 658)*  *It was prepared in accordance with the PRISMA-P statement [47, 48] and PRISMA-NMA checklist [49] (see S1 checklist), guided by Cochrane’s recommendations for systematic review design and execution [44].* |
|  | Comparison list updated in view of improved intervention definitions | *(Line 231)*  *1) Control group (unweaned) versus any form of weaning or separation*  *2) Abrupt weaning versus any alternative weaning intervention*  *3) Comparison between two alternative weaning interventions*  *4) Comparison between management interventions within the same weaning intervention*  *5) Temporary separation with a management intervention aimed at reducing stress*  *6) Any intervention with pre- and post-intervention comparisons* |
