# Supplementary material for: Determining optimal practices for foal weaning – A protocol for a systematic review and network meta-analysis
Source: PLoS One. 2026 Jul 1;21(7):e0352182. doi: 10.1371/journal.pone.0352182 (PMC13322514; doi:10.1371/journal.pone.0352182)
Supplement: Peer review history

# Peer review: Decision Letter 0

**DOI:** 10.1371/journal.pone.0352182.r001

**Authors:** Ewa Tomaszewska

13 Apr 2026

PONE-D-26-12173

Determining optimal practices for foal weaning based on physiological, behavioural, and welfare indices – a protocol for a systematic review and network meta-analysis

PLOS One

Dear Dr. Cranston,

Thank you for submitting your manuscript to PLOS ONE. After careful consideration, we feel that it has merit but does not fully meet PLOS ONE’s publication criteria as it currently stands. Therefore, we invite you to submit a revised version of the manuscript that addresses the points raised during the review process.

Please submit your revised manuscript by May 28 2026 11:59PM. If you will need more time than this to complete your revisions, please reply to this message or contact the journal office at plosone@plos.org. When you're ready to submit your revision, log on to https://www.editorialmanager.com/pone/ and select the 'Submissions Needing Revision' folder to locate your manuscript file.

Please include the following items when submitting your revised manuscript:

- A letter that responds to each point raised by the academic editor and reviewer(s). You should upload this letter as a separate file labeled 'Response to Reviewers'.
- A marked-up copy of your manuscript that highlights changes made to the original version. You should upload this as a separate file labeled 'Revised Manuscript with Track Changes'.
- An unmarked version of your revised paper without tracked changes. You should upload this as a separate file labeled 'Manuscript'.

If you would like to make changes to your financial disclosure, please include your updated statement in your cover letter. Guidelines for resubmitting your figure files are available below the reviewer comments at the end of this letter.

If applicable, we recommend that you deposit your laboratory protocols in protocols.io to enhance the reproducibility of your results. Protocols.io assigns your protocol its own identifier (DOI) so that it can be cited independently in the future. For instructions see: https://journals.plos.org/plosone/s/submission-guidelines#loc-laboratory-protocols. Additionally, PLOS ONE offers an option for publishing peer-reviewed Lab Protocol articles, which describe protocols hosted on protocols.io. Read more information on sharing protocols at https://plos.org/protocols?utm_medium=editorial-email&utm_source=authorletters&utm_campaign=protocols.

As the corresponding author, your ORCID iD is verified in the submission system and will appear in the published article. PLOS supports the use of ORCID, and we encourage all coauthors to register for an ORCID iD and use it as well. Please encourage your coauthors to verify their ORCID iD within the submission system before final acceptance, as unverified ORCID iDs will not appear in the published article. *Only*  the individual author can complete the verification step; PLOS staff *cannot*  verify ORCID iDs on behalf of authors.

We look forward to receiving your revised manuscript.

Kind regards,

Ewa Tomaszewska, DVM Ph.D

Academic Editor

PLOS One

**Journal Requirements:**

When submitting your revision, we need you to address these additional requirements.

1. Please ensure that your manuscript meets PLOS ONE's style requirements, including those for file naming. The PLOS ONE style templates can be found at

https://journals.plos.org/plosone/s/file?id=wjVg/PLOSOne_formatting_sample_main_body.pdf and

https://journals.plos.org/plosone/s/file?id=ba62/PLOSOne_formatting_sample_title_authors_affiliations.pdf

2. We note that the grant information you provided in the ‘Funding Information’ and ‘Financial Disclosure’ sections do not match.

When you resubmit, please ensure that you provide the correct grant numbers for the awards you received for your study in the ‘Funding Information’ section.

3. Please note that funding information should not appear in any section or other areas of your manuscript. We will only publish funding information present in the Funding Statement section of the online submission form. Please remove any funding-related text from the manuscript.

4. Thank you for stating the following in the Competing Interests section:

“I have read the journal's policy and the authors of this manuscript have the following competing interests:

JL is a registered equine breeder with a financial stake in the industry. The remaining authors declare no financial competing interests.

There is a possibility that one or more members of this review team will be co-authors of papers under review. In such instances, the author will not participate in the screening, data extraction, quality assessment, or synthesis of that paper to avoid any conflict of interest.”

Please confirm that this does not alter your adherence to all PLOS ONE policies on sharing data and materials, by including the following statement: "This does not alter our adherence to  PLOS ONE policies on sharing data and materials.” (as detailed online in our guide for authors http://journals.plos.org/plosone/s/competing-interests).  If there are restrictions on sharing of data and/or materials, please state these. Please note that we cannot proceed with consideration of your article until this information has been declared.

Please include your updated Competing Interests statement in your cover letter; we will change the online submission form on your behalf.

5. PLOS requires an ORCID iD for the corresponding author in Editorial Manager on papers submitted after December 6th, 2016. Please ensure that you have an ORCID iD and that it is validated in Editorial Manager. To do this, go to ‘Update my Information’ (in the upper left-hand corner of the main menu), and click on the Fetch/Validate link next to the ORCID field. This will take you to the ORCID site and allow you to create a new iD or authenticate a pre-existing iD in Editorial Manager.

6. We note you have included a table to which you do not refer in the text of your manuscript. Please ensure that you refer to Table 1 in your text; if accepted, production will need this reference to link the reader to the Table.

7. If the reviewer comments include a recommendation to cite specific previously published works, please review and evaluate these publications to determine whether they are relevant and should be cited. There is no requirement to cite these works unless the editor has indicated otherwise.

[Note: HTML markup is below. Please do not edit.]

Reviewers' comments:

Reviewer's Responses to Questions

**Comments to the Author**

1. Does the manuscript provide a valid rationale for the proposed study, with clearly identified and justified research questions?

The research question outlined is expected to address a valid academic problem or topic and contribute to the base of knowledge in the field.

Reviewer #1: Yes

Reviewer #2: Yes

Reviewer #3: Yes

Reviewer #4: Yes

**********

2. Is the protocol technically sound and planned in a manner that will lead to a meaningful outcome and allow testing the stated hypotheses?

The manuscript should describe the methods in sufficient detail to prevent undisclosed flexibility in the experimental procedure or analysis pipeline, including sufficient outcome-neutral conditions (e.g. necessary controls, absence of floor or ceiling effects) to test the proposed hypotheses and a statistical power analysis where applicable. As there may be aspects of the methodology and analysis which can only be refined once the work is undertaken, authors should outline potential assumptions and explicitly describe what aspects of the proposed analyses, if any, are exploratory.

Reviewer #1: Yes

Reviewer #2: Yes

Reviewer #3: Yes

Reviewer #4: Yes

**********

3. Is the methodology feasible and described in sufficient detail to allow the work to be replicable?

Descriptions of methods and materials in the protocol should be reported in sufficient detail for another researcher to reproduce all experiments and analyses. The protocol should describe the appropriate controls, sample size calculations, and replication needed to ensure that the data are robust and reproducible.

Reviewer #1: Yes

Reviewer #2: Yes

Reviewer #3: Yes

Reviewer #4: Yes

**********

4. Have the authors described where all data underlying the findings will be made available when the study is complete?

The PLOS Data policy requires authors to make all data underlying the findings described in their manuscript fully available without restriction, with rare exception, at the time of publication. The data should be provided as part of the manuscript or its supporting information, or deposited to a public repository. For example, in addition to summary statistics, the data points behind means, medians and variance measures should be available. If there are restrictions on publicly sharing data—e.g. participant privacy or use of data from a third party—those must be specified.

Reviewer #1: Yes

Reviewer #2: Yes

Reviewer #3: Yes

Reviewer #4: Yes

**********

5. Is the manuscript presented in an intelligible fashion and written in standard English?

PLOS ONE does not copyedit accepted manuscripts, so the language in submitted articles must be clear, correct, and unambiguous. Any typographical or grammatical errors should be corrected at revision, so please note any specific errors here.

Reviewer #1: Yes

Reviewer #2: Yes

Reviewer #3: Yes

Reviewer #4: Yes

**********

6. Review Comments to the Author

Please use the space provided to explain your answers to the questions above and, if applicable, provide comments about issues authors must address before this protocol can be accepted for publication. You may also include additional comments for the author, including concerns about research or publication ethics.

You may also provide optional suggestions and comments to authors that they might find helpful in planning their study.

(Please upload your review as an attachment if it exceeds 20,000 characters)

Reviewer #1: Scientific Comments on the Review (Point-by-Point Evaluation and Suggested Corrections)

1. Line 77–84: Introduction – Contextualizing Equine Welfare Concerns

Comment:

The opening effectively establishes the societal and ethical context for improving equine welfare, citing relevant literature (e.g., Douglas et al., 2022; Heleski, 2023). However, the claim that "the horse industry faces increasing public pressure" would benefit from more specific data or survey-based evidence to substantiate the trend. Consider citing public opinion studies or reports from animal welfare NGOs (e.g., World Horse Welfare) to strengthen this assertion.

Suggested revision:

"Growing public scrutiny of equine welfare, evidenced by increased media coverage and consumer surveys (e.g., World Horse Welfare, 2023), has placed pressure on the industry..."

2. Line 79–81: Reference to 2021 Olympic Incident

Comment:

While the reference to the 2021 Olympic Pentathlon incident is accurate and impactful, the citation (Cuckson, 2021) appears to refer to a news report rather than a peer-reviewed source. For scientific rigor in a systematic review protocol, it is preferable to cite formal investigations or official reports (e.g., FEI inquiry outcomes) where available.

Suggested revision:

Replace with:

"...such as the 2021 Olympic Modern Pentathlon horse incident (FEI, 2021), which prompted widespread criticism and calls for reform."

3. Line 86–89: Broadening Scope to Weaning Practices

Comment:

This transition is logical and well-supported. The inclusion of Pearson and Douglas (2025) strengthens the argument that weaning falls within broader welfare concerns. However, ensure that this reference is not forward-dated relative to the current date (April 2026); if unpublished or in press, clarify its status appropriately.

4. Line 91–104: Definition and Stress of Weaning

Comment:

The definition of weaning as “nutritional separation” aligns with Latham and Mason (2008) and Henry et al. (2020), but could be expanded slightly to acknowledge behavioural and emotional components of dam-offspring bonding, which contribute to stress responses. This would enhance biological plausibility.

Suggested addition:

"Weaning involves not only nutritional separation but also disruption of a strong social bond, contributing to significant psychological stress in foals."

Also, consider clarifying that abrupt weaning often includes abrupt social separation, which compounds stress beyond nutrition alone.

5. Line 107–117: Heterogeneity in Gradual Weaning Definitions

Comment:

This is a critical and well-articulated point. The lack of standardised definitions for "gradual weaning" across studies significantly undermines comparability. To strengthen this section, explicitly define how your review will categorise gradual vs. abrupt weaning based on operational criteria (e.g., duration of separation phases, frequency of contact).

Suggested clarification in Methods:

Add under Interventions:

"Gradual weaning will be operationally defined as any protocol involving staged physical separation over ≥3 days, including partial-contact systems (e.g., fenceline weaning), while abrupt weaning refers to immediate and complete separation without prior acclimatisation."

6. Line 119–125: Additional Management Strategies

Comment:

The list of strategies (nannies, diet modification, later weaning, pharmacology) is comprehensive. However, grouping pharmacological interventions (e.g., anxiolytics) with management practices may conflate fundamentally different approaches—one modifies physiology, others modify environment. Consider distinguishing between management-based and medical/pharmacological interventions in analysis.

Suggestion:

In Data Items or Subgroups, classify interventions into categories:

• Environmental (housing, conspecifics)

• Temporal (age at weaning, weaning method)

• Dietary (pre-/post-weaning nutrition)

• Medical (pharmaceutical use)

This improves interpretability in NMA.

7. Line 130–140: Long-Term Consequences of Weaning Stress

Comment:

The paragraph convincingly links early-life stress to long-term developmental impacts. However, the citation of Valenchon et al. (2025) again raises concern about temporal validity (assuming current date is April 2026). If this work has not yet been published, it should be cited as "in press" or removed until verified.

Additionally, consider adding a sentence linking these long-term effects directly to economic and performance implications for breeders, enhancing translational relevance.

8. Line 156–165: Justification for Systematic Review and NMA

Comment:

Strong justification for using both pairwise and network meta-analysis. However, note that Bayesian NMA requires careful handling of multi-arm trials and assumptions about independence. Clarify whether multi-arm studies will be accounted for using appropriate methods (e.g., arm-based or contrast-based models with covariance adjustment).

Suggested addition in Methods:

"For multi-arm trials, correlations between effect estimates will be accounted for using arm-based models or variance-correction techniques to avoid unit-of-analysis errors."

9. Line 219–222: Timeline of Review Process

Comment:

Given that full-text screening is expected by July 2026 and submission by late 2027, the statement appears plausible. However, since the initial search was conducted in August 2025, confirm that a study update search will be performed immediately before final synthesis (as noted in line 339), particularly given the two-year gap.

Ensure this is clearly stated to maintain currency.

10. Line 240–261: Intervention Criteria

Comment:

Clear distinction between planned and unplanned weaning is appropriate. However, the exclusion of wild/feral populations is justified but could be nuanced—some feral studies (e.g., on mustangs or brumbies) provide valuable baseline data on natural weaning patterns. While excluded from quantitative synthesis, consider mentioning their value in supplementary discussion.

Reviewer #2: Review Comments to the Author

This manuscript presents a timely and relevant systematic review and network meta-analysis protocol on foal weaning practices. The study demonstrates strong methodological rigor, transparency, and adherence to PRISMA-P guidelines, with strengths including OSF registration, grey literature inclusion, and planned pairwise and Bayesian NMA.

However, several issues should be addressed before publication.

Major concerns:

The inclusion of diverse study designs (e.g., observational and case series) may introduce heterogeneity and bias; further justification and clarification of their role in synthesis are needed. The use of the Cochrane RoB v1 tool should be justified or updated to RoB 2. Outcome heterogeneity requires clearer strategies for harmonisation and prioritisation. The assumptions underlying NMA, particularly transitivity, need stronger justification and explanation of how violations will be handled. Additionally, the choice of Bayesian priors should be justified and supported with references.

Minor concerns:

The Introduction could be more concise, and tense should consistently reflect a protocol. The Google Scholar search limit requires justification. Consider reporting inter-rater reliability, clarifying subgroup definitions, and acknowledging limitations related to publication bias and language restrictions.

Overall, this is a well-designed and impactful protocol. With revisions to improve methodological clarity, it will be suitable for publication.

Recommendation: Major Revision

Reviewer #3: Major comments

1.Title: Shorten the title for readability;

2. Abstract: Clarify the type of study (protocol only) and explicitly state that no empirical results are included.

3.Confirm the OSF registration DOI (https://doi.org/10.17605/OSF.IO/DJWQY) is active and publicly accessible. Add a sentence confirming no protocol amendments beyond those already documented.

4.Standardize terminology: abrupt weaning and gradual weaning definitions are clear but should be formally defined in a single box or paragraph for consistency.

5.Explicitly state whether language is restricted to English (currently implied but not clearly stated).

6. Methods: Specify the exact six databases used (listed in Table 1 but not fully listed in the main text); In NMA: Clearly state the reference intervention (abrupt individual weaning) earlier in Methods; Define time-frame categories (immediate/short/medium/long-term post-weaning) in a table for quick reference.

Minor comments

1.Add a brief graphical abstract or flow diagram of review stages for readability.

2.Include a list of predefined primary/secondary outcomes in a table.

3.Specify planned software versions (R, RevMan, Covidence) if known.

Reviewer #4: It is a very detailed protocol for a systematic review and network meta-analysis. I do not see the need for any amendments.

**********

7. PLOS authors have the option to publish the peer review history of their article (what does this mean?). If published, this will include your full peer review and any attached files.

If you choose “no”, your identity will remain anonymous but your review may still be made public.

**Do you want your identity to be public for this peer review?**  For information about this choice, including consent withdrawal, please see our Privacy Policy.

Reviewer #1: No

Reviewer #2: No

Reviewer #3: No

Reviewer #4: No

**********

[NOTE: If reviewer comments were submitted as an attachment file, they will be attached to this email and accessible via the submission site. Please log into your account, locate the manuscript record, and check for the action link "View Attachments". If this link does not appear, there are no attachment files.]

To ensure your figures meet our technical requirements, please review our figure guidelines: https://journals.plos.org/plosone/s/figures

You may also use PLOS’s free figure tool, NAAS, to help you prepare publication quality figures: https://journals.plos.org/plosone/s/figures#loc-tools-for-figure-preparation.

NAAS will assess whether your figures meet our technical requirements by comparing each figure against our figure specifications.

Attachment Submitted filename: Scientific Comments on the Review.docx

# Author comment: Author response to Decision Letter 1

**DOI:** 10.1371/journal.pone.0352182.r002

27 May 2026

Reviewer Comment Action

Journal 1. Please ensure that your manuscript meets PLOS ONE's style requirements, including those for file naming. The PLOS ONE style templates can be found at

https://journals.plos.org/plosone/s/file?id=wjVg/PLOSOne_formatting_sample_main_body.pdf and

https://journals.plos.org/plosone/s/file?id=ba62/PLOSOne_formatting_sample_title_authors_affiliations.pdf

The manuscript has been reformatted as per PLOS One style requirements, in accordance with the attached guidelines.

Journal 2. We note that the grant information you provided in the ‘Funding Information’ and ‘Financial Disclosure’ sections do not match.

When you resubmit, please ensure that you provide the correct grant numbers for the awards you received for your study in the ‘Funding Information’ section. Thank you, we have added an updated version to the cover letter as requested.

Journal 3. Please note that funding information should not appear in any section or other areas of your manuscript. We will only publish funding information present in the Funding Statement section of the online submission form. Please remove any funding-related text from the manuscript. Funding related information has been removed from the manuscript and will only be reported within the Funding Statement section on submission.

Journal 4. Thank you for stating the following in the Competing Interests section:

“I have read the journal's policy and the authors of this manuscript have the following competing interests:

JL is a registered equine breeder with a financial stake in the industry. The remaining authors declare no financial competing interests.

There is a possibility that one or more members of this review team will be co-authors of papers under review. In such instances, the author will not participate in the screening, data extraction, quality assessment, or synthesis of that paper to avoid any conflict of interest.”

Please confirm that this does not alter your adherence to all PLOS ONE policies on sharing data and materials, by including the following statement: "This does not alter our adherence to PLOS ONE policies on sharing data and materials.” (as detailed online in our guide for authors http://journals.plos.org/plosone/s/competing-interests). If there are restrictions on sharing of data and/or materials, please state these. Please note that we cannot proceed with consideration of your article until this information has been declared.

Please include your updated Competing Interests statement in your cover letter; we will change the online submission form on your behalf.

The cover letter has been updated to reflect the competing interests statement, with the requested appropriate acknowledgement.

Journal 5. PLOS requires an ORCID iD for the corresponding author in Editorial Manager on papers submitted after December 6th, 2016. Please ensure that you have an ORCID iD and that it is validated in Editorial Manager. To do this, go to ‘Update my Information’ (in the upper left-hand corner of the main menu), and click on the Fetch/Validate link next to the ORCID field. This will take you to the ORCID site and allow you to create a new iD or authenticate a pre-existing iD in Editorial Manager ORCID link validated in NC's profile

Journal 6. We note you have included a table to which you do not refer in the text of your manuscript. Please ensure that you refer to Table 1 in your text; if accepted, production will need this reference to link the reader to the Table. The references to all tables have been checked to ensure they are cited.

Journal 7. If the reviewer comments include a recommendation to cite specific previously published works, please review and evaluate these publications to determine whether they are relevant and should be cited. There is no requirement to cite these works unless the editor has indicated otherwise. Suggested references were reviewed for relevance, and appropriate citations have been incorporated where relevant.

Reviewer 1 1. Line 77–84: Introduction – Contextualizing Equine Welfare Concerns

Comment:

The opening effectively establishes the societal and ethical context for improving equine welfare, citing relevant literature (e.g., Douglas et al., 2022; Heleski, 2023).

However, the claim that "the horse industry faces increasing public pressure" would benefit from more specific data or survey-based evidence to substantiate the trend. Consider citing public opinion studies or reports from animal welfare NGOs (e.g., World Horse Welfare) to strengthen this assertion.

Suggested revision:

"Growing public scrutiny of equine welfare, evidenced by increased media coverage and consumer surveys (e.g., World Horse Welfare, 2023), has placed pressure on the industry..." Thank you for the suggestion. We have incorporated the suggestion, including additional public survey references.

(Line 49)

The horse industry faces increasing public pressure to improve equine welfare [1, 2]. The growing public scrutiny of equine welfare, evidenced by increased media coverage and consumer surveys [3, 4] have placed pressure on the industry to improve the ethical treatment of horses (Equus caballus) [2].

[3] Thoroughbred Welfare Initiative, 2021

[4] Equine Ethics and Wellbeing Commission (FEI), 2022)

Reviewer 1 2. Line 79–81: Reference to 2021 Olympic Incident

Comment:

While the reference to the 2021 Olympic Pentathlon incident is accurate and impactful, the citation (Cuckson, 2021) appears to refer to a news report rather than a peer-reviewed source. For scientific rigor in a systematic review protocol, it is preferable to cite formal investigations or official reports (e.g., FEI inquiry outcomes) where available.

Suggested revision:

Replace with:

"...such as the 2021 Olympic Modern Pentathlon horse incident (FEI, 2021), which prompted widespread criticism and calls for reform."

We believe the previously suggested revision has addressed this concern.

Reviewer 1 3. Line 86–89: Broadening Scope to Weaning Practices

Comment:

This transition is logical and well-supported. The inclusion of Pearson and Douglas (2025) strengthens the argument that weaning falls within broader welfare concerns.

However, ensure that this reference is not forward-dated relative to the current date (April 2026); if unpublished or in press, clarify its status appropriately. This reference is published in the Equine Veterinary Journal and therefore no change was required.

(Line 668)

Pearson G, Douglas J. Start ‘em young, treat ‘em right: How horses’ early life experiences can set them up for success in life. Equine Veterinary Journal. 2025;57(3):540-5. doi: 10.1111/evj.14485.

Reviewer 1 4. Line 91–104: Definition and Stress of Weaning

Comment:

The definition of weaning as “nutritional separation” aligns with Latham and Mason (2008) and Henry et al. (2020), but could be expanded slightly to acknowledge behavioural and emotional components of dam-offspring bonding, which contribute to stress responses. This would enhance biological plausibility.

Suggested addition:

"Weaning involves not only nutritional separation but also disruption of a strong social bond, contributing to significant psychological stress in foals."

Also, consider clarifying that abrupt weaning often includes abrupt social separation, which compounds stress beyond nutrition alone.

The original text was retained to maintain the accuracy of the definition of the term weaning, with the suggested additions incorporated later in the paragraph.

(Change at line 69)

Weaning is recognised as one of the most stressful events in a horse's life [9-12]. As with other mammals, weaning is defined as the nutritional separation from the mare [11, 13]. In naturalistic settings, weaning usually occurs spontaneously around nine months of age, with foals remaining with their maternal herd until they are two to three years old [11]. In domestic settings, however, weaning is usually artificially implemented and generally occurs between four and eight months of age [9-11, 14, 15]. In addition to nutritional separation, weaning also involves complete physical, visual, and auditory separation of the foal from the mare. The splitting of the mare and foal dyad is defined as either abrupt weaning, due to immediate separation, or gradual weaning if separation occurs in stages. Weaning may also occur in an unfamiliar environment and often involves individual foals kept in complete isolation. Domestic weaning therefore involves not only nutritional separation but also the disruption of strong social bonds, contributing to significant psychological stress in foals, which is further compounded in those weaned abruptly.

Reviewer 1 5. Line 107–117: Heterogeneity in Gradual Weaning Definitions

Comment:

This is a critical and well-articulated point. The lack of standardised definitions for "gradual weaning" across studies significantly undermines comparability. To strengthen this section, explicitly define how your review will categorise gradual vs. abrupt weaning based on operational criteria (e.g., duration of separation phases, frequency of contact).

Suggested clarification in Methods:

Add under Interventions:

"Gradual weaning will be operationally defined as any protocol involving staged physical separation over ≥3 days, including partial-contact systems (e.g., fenceline weaning), while abrupt weaning refers to immediate and complete separation without prior acclimatisation."

Thank you for the feedback. The text has been revised to reflect the reviewers' suggestion.

(Methods/Intervention section - Line 188)

Abrupt weaning will be characterised as the immediate and complete separation of the foal from the dam without prior acclimatisation. This may occur individually (reference intervention), in pairs or in groups of foals, but without the presence of other adults. The reference intervention will be compared to alternative weaning interventions, including:

1) Abrupt weaning with a nanny (adult horse): foal weaned abruptly from the dam but left in the care of another adult horse;

2) Progressive removal of mares: progressive removal of 1-2 dams from an established dam-foal herd through the abrupt weaning of the foal over ≥ 2 days until all mares have been removed;

3) Gradual acclimatisation: foals gradually acclimatised to separations prior to (≥ 2 weeks) the commencement of the weaning process;

4) Gradual partial contact: partial contact maintained for ≥ 2 days prior to complete separation (e.g. fenceline, nutritional separation via covering udder);

5) Gradual over time: increasing separation time ≥ 2 days prior to complete separation;

6) Gradual over distance: increasing the distance separated over ≥ 2 days prior to complete separation; and

7) Other: any other weaning intervention not covered by previous definitions.

Reviewer 1 6. Line 119–125: Additional Management Strategies

Comment:

The list of strategies (nannies, diet modification, later weaning, pharmacology) is comprehensive. However, grouping pharmacological interventions (e.g., anxiolytics) with management practices may conflate fundamentally different approaches—one modifies physiology, others modify environment. Consider distinguishing between management-based and medical/pharmacological interventions in analysis.

Suggestion:

In Data Items or Subgroups, classify interventions into categories:

• Environmental (housing, conspecifics)

• Temporal (age at weaning, weaning method)

• Dietary (pre-/post-weaning nutrition)

• Medical (pharmaceutical use)

This improves interpretability in NMA.

Clarification on the definitions of "management intervention" has been provided in the Methods to distinguish between factors treated as study interventions versus those analysed as subgroup/moderating variables. In addition, management-based interventions have been categorised in line with the reviewers' suggestion .

(Methods/Interventions - Line 208)

Any additional management strategy specifically explored at the study treatment level, while keeping the primary weaning intervention constant, will be classified as a management intervention. The management interventions will be categorised as:

• Environmental (housing, conspecifics, handling)

• Temporal (age at weaning)

• Dietary (pre/post-weaning nutrition)

• Medical (pharmaceutical use).

However, when these factors are observed but not evaluated as a treatment-level comparison, they will be considered study-level characteristics and explored as subgroup or moderating factors. Additionally, experiments reporting temporary separations of the target population from their dam, with a management intervention aimed at reducing stress during the separation will also be included.

Reviewer 1 7. Line 130–140: Long-Term Consequences of Weaning Stress

Comment:

The paragraph convincingly links early-life stress to long-term developmental impacts. However, the citation of Valenchon et al. (2025) again raises concern about temporal validity (assuming current date is April 2026). If this work has not yet been published, it should be cited as "in press" or removed until verified.

This reference has now been published in Nature Communications, and the reference has therefore been updated accordingly.

(Line 747)

Valenchon M, Reigner F, Lefort G, Adriaensen H, Gesbert A, Barrière P, et al. Affiliative behaviours regulate allostasis development and shape biobehavioural trajectories in horses. Nature Communications. 2026;17(1):47. doi: 10.1038/s41467-025-66729-1.

Reviewer 1 Additionally, consider adding a sentence linking these long-term effects directly to economic and performance implications for breeders, enhancing translational relevance.

Thank you for the suggestion, a linking sentence has been added.

(Line 97)

This, in turn, can affect learning ability, fearfulness, and handling efficiency which influence performance, and ultimately the commercial value of the horse [37-41]. Therefore, it is in the industry’s best interest to adopt weaning practices that safeguard foal welfare.

Reviewer 1 8. Line 156–165: Justification for Systematic Review and NMA

Comment:

Strong justification for using both pairwise and network meta-analysis. However, note that Bayesian NMA requires careful handling of multi-arm trials and assumptions about independence. Clarify whether multi-arm studies will be accounted for using appropriate methods (e.g., arm-based or contrast-based models with covariance adjustment).

Suggested addition in Methods:

"For multi-arm trials, correlations between effect estimates will be accounted for using arm-based models or variance-correction techniques to avoid unit-of-analysis errors."

Great feedback, thank you. The text has been revised to reflect the reviewer's suggestion.

(Methods/Effect measurements - Line 436)

For multi-arm trials, correlations between effect estimates will be accounted for using arm-based models or variance-correction techniques to avoid unit-of-analysis errors.

Reviewer 1 9. Line 219–222: Timeline of Review Process

Comment:

Given that full-text screening is expected by July 2026 and submission by late 2027, the statement appears plausible. However, since the initial search was conducted in August 2025, confirm that a study update search will be performed immediately before final synthesis (as noted in line 339), particularly given the two-year gap.

Ensure this is clearly stated to maintain currency.

Thank you, the text has been updated. The protocol now specifies that the search will be repeated immediately prior to the data synthesis stage to capture any new published studies.

(Line 296)

The search will be repeated immediately prior to data synthesis to identify any new research and again if the review is not published within 12 months of the previous search. Any studies published after this time will be noted but excluded from the synthesis.

Reviewer 1 10. Line 240–261: Intervention Criteria

Comment:

Clear distinction between planned and unplanned weaning is appropriate. However, the exclusion of wild/feral populations is justified but could be nuanced—some feral studies (e.g., on mustangs or brumbies) provide valuable baseline data on natural weaning patterns. While excluded from quantitative synthesis, consid

Attachment Submitted filename: Response_to_Reviewers.docx

# Peer review: Decision Letter 1

**DOI:** 10.1371/journal.pone.0352182.r003

**Authors:** Ewa Tomaszewska

8 Jun 2026

Determining optimal practices for foal weaning – a protocol for a systematic review and network meta-analysis

PONE-D-26-12173R1

Dear Dr. Nicole Cranston,

We’re pleased to inform you that your manuscript has been judged scientifically suitable for publication and will be formally accepted for publication once it meets all outstanding technical requirements.

Within one week, you’ll receive an e-mail detailing the required amendments. When these have been addressed, you’ll receive a formal acceptance letter and your manuscript will be scheduled for publication.

An invoice will be generated when your article is formally accepted. Please note, if your institution has a publishing partnership with PLOS and your article meets the relevant criteria, all or part of your publication costs will be covered. Please make sure your user information is up-to-date by logging into Editorial Manager at Editorial Manager® and clicking the ‘Update My Information' link at the top of the page. For questions related to billing, please contact billing support.

If your institution or institutions have a press office, please notify them about your upcoming paper to help maximize its impact. If they’ll be preparing press materials, please inform our press team as soon as possible -- no later than 48 hours after receiving the formal acceptance. Your manuscript will remain under strict press embargo until 2 pm Eastern Time on the date of publication. For more information, please contact onepress@plos.org.

Kind regards,

Ewa Tomaszewska, DVM Ph.D

Academic Editor

PLOS One

Additional Editor Comments (optional):

Reviewers' comments:

Reviewer's Responses to Questions

**Comments to the Author**

1. Does the manuscript provide a valid rationale for the proposed study, with clearly identified and justified research questions?

The research question outlined is expected to address a valid academic problem or topic and contribute to the base of knowledge in the field.

Reviewer #2: Yes

Reviewer #3: Yes

**********

2. Is the protocol technically sound and planned in a manner that will lead to a meaningful outcome and allow testing the stated hypotheses?

The manuscript should describe the methods in sufficient detail to prevent undisclosed flexibility in the experimental procedure or analysis pipeline, including sufficient outcome-neutral conditions (e.g. necessary controls, absence of floor or ceiling effects) to test the proposed hypotheses and a statistical power analysis where applicable. As there may be aspects of the methodology and analysis which can only be refined once the work is undertaken, authors should outline potential assumptions and explicitly describe what aspects of the proposed analyses, if any, are exploratory.

Reviewer #2: Yes

Reviewer #3: Yes

**********

3. Is the methodology feasible and described in sufficient detail to allow the work to be replicable?

Descriptions of methods and materials in the protocol should be reported in sufficient detail for another researcher to reproduce all experiments and analyses. The protocol should describe the appropriate controls, sample size calculations, and replication needed to ensure that the data are robust and reproducible.

Reviewer #2: Yes

Reviewer #3: Yes

**********

4. Have the authors described where all data underlying the findings will be made available when the study is complete?

The PLOS Data policy requires authors to make all data underlying the findings described in their manuscript fully available without restriction, with rare exception, at the time of publication. The data should be provided as part of the manuscript or its supporting information, or deposited to a public repository. For example, in addition to summary statistics, the data points behind means, medians and variance measures should be available. If there are restrictions on publicly sharing data—e.g. participant privacy or use of data from a third party—those must be specified.

Reviewer #2: Yes

Reviewer #3: Yes

**********

5. Is the manuscript presented in an intelligible fashion and written in standard English?

PLOS ONE does not copyedit accepted manuscripts, so the language in submitted articles must be clear, correct, and unambiguous. Any typographical or grammatical errors should be corrected at revision, so please note any specific errors here.

Reviewer #2: Yes

Reviewer #3: Yes

**********

6. Review Comments to the Author

Please use the space provided to explain your answers to the questions above and, if applicable, provide comments about issues authors must address before this protocol can be accepted for publication. You may also include additional comments for the author, including concerns about research or publication ethics.

You may also provide optional suggestions and comments to authors that they might find helpful in planning their study.

(Please upload your review as an attachment if it exceeds 20,000 characters)

Reviewer #2: The protocol addresses an important and timely topic in equine welfare, namely the identification and comparison of artificial weaning practices that may reduce stress and improve welfare outcomes in foals. The research questions are clearly stated and justified, and the protocol is generally well designed, methodologically rigorous, and aligned with current best-practice guidance for systematic reviews and network meta-analyses. Strengths of the protocol include a comprehensive search strategy, clearly defined eligibility criteria, independent screening and data extraction procedures, appropriate risk-of-bias assessment methods, and detailed plans for both pairwise meta-analysis and Bayesian network meta-analysis. The authors also appropriately consider important assumptions underlying network meta-analysis, including heterogeneity, transitivity, and coherence, and have proposed sensitivity analyses to assess the robustness of findings. The planned use of GRADE and CINeMA frameworks further strengthens the assessment of evidence certainty. The manuscript is generally well written, logically structured, and presented in clear academic English. The methods are described in sufficient detail to allow replication by other researchers, and the commitment to making study materials available through the Open Science Framework is commendable. I have only a few minor suggestions for improvement. First, the authors may wish to provide additional detail regarding which datasets, extracted data files, analytical code, and supplementary materials will be deposited in the OSF repository to further strengthen compliance with data-sharing requirements. Second, although formal power calculations are not typically feasible for systematic reviews and network meta-analyses because the available evidence base is unknown at the protocol stage, a brief acknowledgment of this limitation within the methods section would be helpful. Third, given the anticipated heterogeneity in weaning interventions and outcome measurements, the authors may consider providing additional justification for the planned categorisation of moderators and subgroup definitions.

Overall, this is a high-quality protocol addressing a relevant research question. The proposed methodology is appropriate, feasible, and likely to produce meaningful findings that will contribute to the evidence base on equine welfare and weaning management practices. Subject to minor revisions and clarifications, I believe the protocol is suitable for publication.

Reviewer #3: The authors had revised the manuscript according to reviewer's comments. The current vesion meet the standard of Plos One.

**********

7. PLOS authors have the option to publish the peer review history of their article (what does this mean?). If published, this will include your full peer review and any attached files.

If you choose “no”, your identity will remain anonymous but your review may still be made public.

**Do you want your identity to be public for this peer review?**  For information about this choice, including consent withdrawal, please see our Privacy Policy.

Reviewer #2: No

Reviewer #3: No

**********

# Editor report: Acceptance letter

**DOI:** 10.1371/journal.pone.0352182.r004

**Authors:** Ewa Tomaszewska

PONE-D-26-12173R1

PLOS One

Dear Dr. Cranston,

I'm pleased to inform you that your manuscript has been deemed suitable for publication in PLOS One. Congratulations! Your manuscript is now being handed over to our production team.

At this stage, our production department will prepare your paper for publication. This includes ensuring the following:

* All references, tables, and figures are properly cited

* All relevant supporting information is included in the manuscript submission,

* There are no issues that prevent the paper from being properly typeset

You will receive further instructions from the production team, including instructions on how to review your proof when it is ready. Please keep in mind that we are working through a large volume of accepted articles, so please give us a few days to review your paper and let you know the next and final steps.

Lastly, if your institution or institutions have a press office, please let them know about your upcoming paper now to help maximize its impact. If they'll be preparing press materials, please inform our press team within the next 48 hours. Your manuscript will remain under strict press embargo until 2 pm Eastern Time on the date of publication. For more information, please contact onepress@plos.org.

You will receive an invoice from PLOS for your publication fee after your manuscript has reached the completed accept phase. If you receive an email requesting payment before acceptance or for any other service, this may be a phishing scheme. Learn how to identify phishing emails and protect your accounts at https://explore.plos.org/phishing.

If we can help with anything else, please email us at customercare@plos.org.

Thank you for submitting your work to PLOS ONE and supporting open access.

Kind regards,

PLOS ONE Editorial Office Staff

on behalf of

Professor Ewa Tomaszewska

Academic Editor

PLOS One
